# IL-17/CXCL5 signaling within the oligovascular niche mediates human and mouse white matter injury

**DOI:** 10.1016/j.celrep.2022.111848

**Published:** 2022-12-20

**Authors:** Guanxi Xiao, Rosie Kumar, Yutaro Komuro, Jasmine Burguet, Visesha Kakarla, Ida Azizkhanian, Sunil A. Sheth, Christopher K. Williams, Xinhai R. Zhang, Michal Macknicki, Andrew Brumm, Riki Kawaguchi, Phu Mai, Naoki Kaneko, Harry V. Vinters, S. Thomas Carmichael, Leif A. Havton, Charles DeCarli, Jason D. Hinman

**Affiliations:** 1 Department of Neurology, David Geffen School of Medicine, University of California, Los Angeles, Los Angeles, CA, USA; 2 Institut Jean-Pierre Bourgin, INRA, AgroParisTech, CNRS, Université Paris-Saclay, 78000 Versailles, France; 3 New York Medical College, School of Medicine, Valhalla, NY, USA; 4 Department of Neurology, UT Health McGovern School of Medicine, Houston, TX, USA; 5 Department of Neuropathology, David Geffen School of Medicine, University of California, Los Angeles, Los Angeles, CA, USA; 6 Department of Psychiatry, Semel Institute for Neuroscience and Human Behavior, University of California, Los Angeles, Los Angeles, CA, USA; 7 Department of Radiological Sciences, David Geffen School of Medicine, University of California, Los Angeles, Los Angeles, CA, USA; 8 Department of Neurobiology, David Geffen School of Medicine, University of California, Los Angeles, Los Angeles, CA, USA; 9 Department of Neurology, University of California, Davis, Davis, CA, USA; 10 Twitter: @hinmanlabUCLA; 11 Lead contact

## Abstract

Cerebral small vessel disease and brain white matter injury are worsened by cardiovascular risk factors including obesity. Molecular pathways in cerebral endothelial cells activated by chronic cerebrovascular risk factors alter cell-cell signaling, blocking endogenous and post-ischemic white matter repair. Using cell-specific translating ribosome affinity purification (RiboTag) in white matter endothelia and oligodendrocyte progenitor cells (OPCs), we identify a coordinated interleukin-chemokine signaling cascade within the oligovascular niche of subcortical white matter that is triggered by diet-induced obesity (DIO). DIO induces interleukin-17B (IL-17B) signaling that acts on the cerebral endothelia through IL-17Rb to increase both circulating and local endothelial expression of CXCL5. In white matter endothelia, CXCL5 promotes the association of OPCs with the vasculature and triggers OPC gene expression programs regulating cell migration through chemokine signaling. Targeted blockade of IL-17B reduced vessel-associated OPCs by reducing endothelial CXCL5 expression. In multiple human cohorts, blood levels of CXCL5 function as a diagnostic and prognostic biomarker of vascular cognitive impairment.

## In brief

Xiao et al. demonstrate that a high-fat diet disrupts brain white matter and exacerbates the response to a subcortical ischemic stroke by 30%. Using cell-specific gene expression studies, they show that this occurs through dysregulated immune signaling between blood vessels and oligodendrocyte progenitor cells acting through IL-17/CXCL5 signaling.

## Graphical Abstract

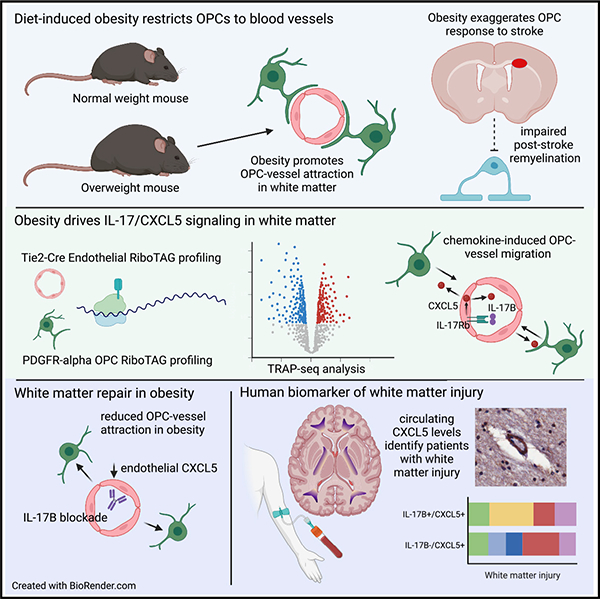

## INTRODUCTION

Cerebral small vessel disease is an age-related entity affecting brain white matter. The resulting white matter lesions accumulate over time^1^ and contribute to disability,^2^ dementia,^3–5^ and death.^6^ Cerebral small vessel injury is significantly worsened by chronic cardiovascular risk factors such as hypertension, diabetes, and obesity.^7–10^ In particular, abdominal obesity and its associated metabolic disturbances in blood pressure, lipids, and blood sugar control increase the risk of developing white matter lesions on magnetic resonance imaging (MRI)^11–14^ and increase the likelihood of lacunar brain infarction or stroke.^15^ While the pathologic changes associated with cerebral small vessel disease are well known,^16,17^ the molecular pathways that drive small vessel injury in the brain are largely unknown.

Emerging data suggest that an interaction between cerebral vessels and cells of the oligodendrocyte lineage play a key role in maintaining white matter homeostasis.^18–20^ A subset of platelet-derived growth factor receptor alpha-positive (PDGFRα+) oligodendrocyte progenitor cells (OPCs) closely associate with the vasculature^21,22^ and use it to migrate in the brain during development.^23^ Proteins secreted by endothelial cells promote OPC migration and proliferation *in vitro*.^24,25^ In the spontaneously hypertensive rat model of cerebral small vessel disease, the OPC population is increased in association with vascular changes, and delays in OPC maturation may be mediated by endothelial secretion of *HSP90α*.^26^ Both the diagnosis and treatment of cerebral small vessel disease would be advanced by identifying additional molecular pathways active in cerebral endothelia and driven by chronic cardiovascular risk factors.^27^

To identify molecular pathways active in the oligovascular niche triggered by chronic cardiovascular risk factors, we used a mouse model of diet-induced obesity (DIO)^28^ that recapitulates a number of features of human cardiovascular risk.^29^ We combined this DIO model with a mouse model of subcortical white matter stroke that mimics human lacunar stroke.^30,31^ In this combined DIO-stroke model, we show that stroke-responsive OPCs are more numerous and persistent after stroke and that post-stroke white matter repair is compromised by DIO. We then use cell-specific translating ribosome affinity purification and RNA sequencing in Tie2-Cre:RiboTag and PDGFRα-CreERT2:RiboTag mice to identify the oligovascular transcriptome after the onset of DIO. This approach led to the identification of an oligovascular signaling cascade acting through the interleukin-17B (IL-17B)/IL-17 receptor b (Rb) isoforms of the IL-17 family in chronically injured cerebral endothelial cells to increase endothelial CXCL5, which can exert paracrine signaling on OPCs. We hypothesized that this coordinated intercellular signaling cascade could drive endothelial-OPC interactions before and after stroke. Further, we speculated that this DIO-induced signaling cascade could act as a functional biomarker for human cerebral small vessel disease and vascular cognitive impairment. Here, we present evidence that IL-17B/IL-17Rb/CXCL5 signaling is activated by a recognized chronic cerebrovascular risk factor, drives intercellular signaling within the oligovascular niche, and marks a subset of human subjects at risk for vascular cognitive impairment. These findings have direct implications for the understanding of human cerebral small vessel disease.

## RESULTS

### DIO damages white matter microvasculature and promotes endothelial-OPC interactions

Obesity is a significant risk factor for the development of small vessel disease and white matter injury.^10,11,13,14^ We used a well-established model of DIO^28^ to model the effects of chronic cardiovascular risk on brain white matter and the vasculature using Tie2-Cre;tdTomato (Ai14) transgenic mice. After 12 weeks on the dietary intervention, mice on control-fat diet (CFD) gained 5.84 ± 0.78 g, while mice on high-fat diet (HFD) gained 23.2 ± 0.76 g, corresponding to a 71% relative weight gain (p = 0.0004). HFD mice also exhibited metabolic disturbances in cholesterol and blood sugar (Figure S1) consistent with the diagnostic criteria for metabolic syndrome.^32^ Similar changes in weight were induced by DIO in several other transgenic strains used in this study. At 20 weeks of age after the development of obesity, we examined the vasculature and cellular makeup of the white matter.

**Figure 1.**
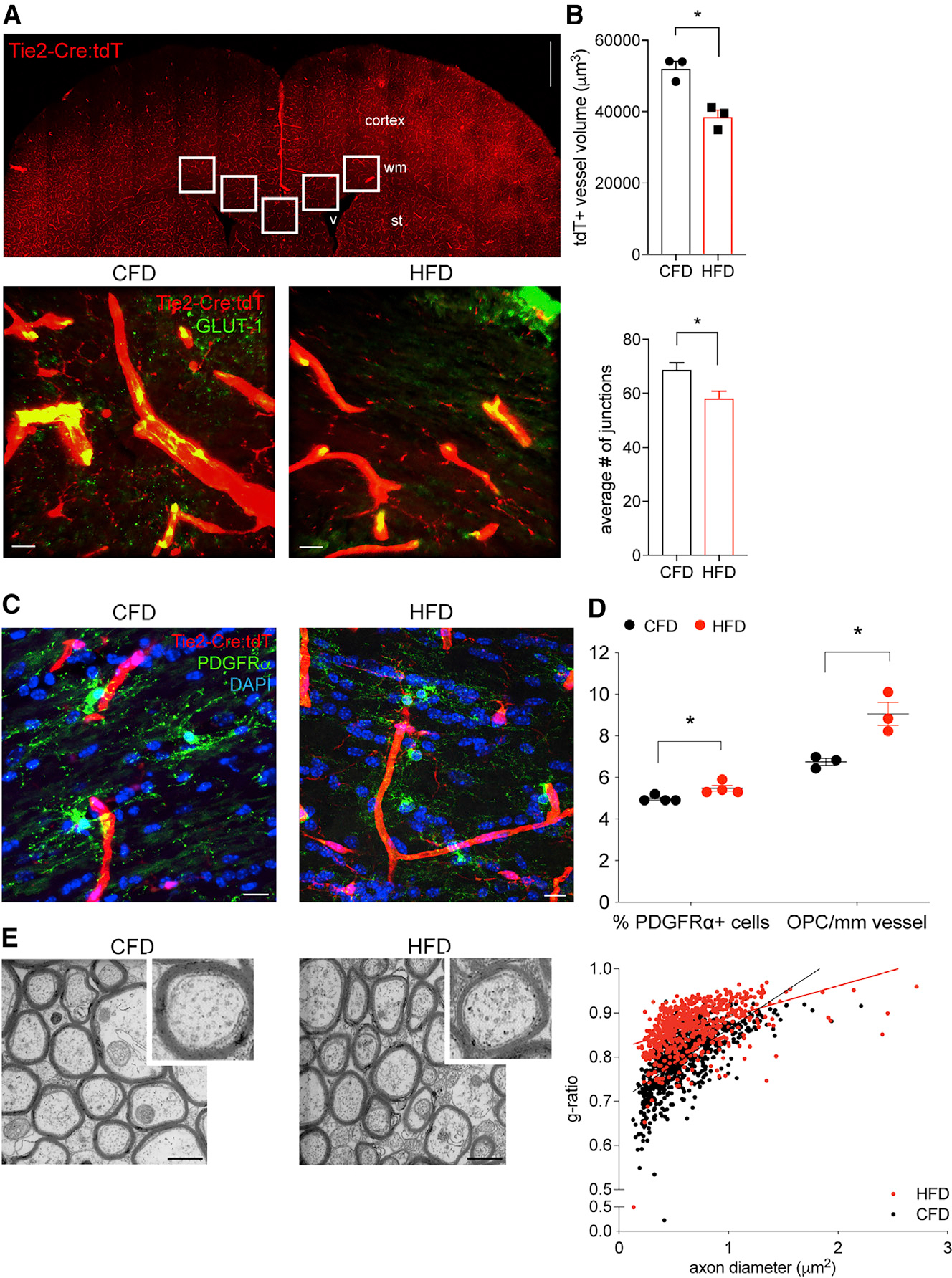
Diet-induced obesity damages white matter microvasculature and promotes endothelial-oligodendrocyte progenitor cell interactions Tie2-Cre;tdTomato transgenic mice (n = 3/grp; 15 confocal z stacks per animal; inset boxes) were used to measure vascular changes after diet-induced obesity (DIO; top). Images of subcortical white matter from CFD (left) and HFD (right) animals labeled for Tie2-Cre;tdTomato (red) and GLUT-1 (green) (A). Average white matter tdT+ vessel volume in CFD (black) and HFD (red) animals (p = 0.0069; top) and average vascular junctions (p = 0.0032; bottom) (B). Vessel-associated PDGFRα+ OPCs in CFD (left) and HFD (right) (C). Percentage of PDGFRα+ OPCs/DAPI+ cells is increased in HFD (5.01% ± 0.13% versus 5.66% ± 0.22%; *p = 0.014) and the number of PDGFRα+ OPCs per mm vessel length is increased (6.46 ± 0.23 versus 8.94 ± 0.31 cells/mm; *p < 0.0001) (D). Representative electron microscopy of the midline sagittal corpus callosum in CFD (left) and HFD (right) animals at 20 weeks of age (n = 6/grp). Distribution of axon diameter versus g ratio in animals on CFD (black) versus HFD (red) demonstrates an increased average g ratio (0.88 versus 0.80; **p = 0.002) in animals on HFD compared with CFD (E). Scalebars: 500 μm (A), 10 μm (BandC), and 1 μm (E).

In Tie2-Cre;tdTomato (Ai14) transgenic mice, DIO reduces the volume of tdTomato (tdT)+ vessels and the branch complexity of the vasculature within subcortical white matter (26%, p = 0.0069, and 15.4%, p = 0.0032, respectively) (Figures 1A and 1B). In addition to the DIO-induced reduction in white matter microvasculature, we also observed an increase in the percentage of PDGFRα+ OPCs within the corpus callosum (PDGFRα+/DAPI+, 5.01% ± 0.13% versus 5.66 ± 0.22%; p = 0.014) and a concordant increase in OPCs associated with vessels, measured as OPCs per unit vessel length (6.46 ± 0.23 versus 8.94 ± 0.31 cells/mm; p < 0.0001) (Figures 1C and 1D). Notably, DIO also appeared to alter the morphology of OPCs from a predominantly stellate morphology to an intermediate and/or perivascular cell type as reported by Kishida et al.^21^ This change in OPCs occurs in the absence of difference in the percentage of GST-π-+ mature oligodendrocytes (Figures S2A and S2B) but is associated with thinner myelin sheaths and an increase in the average g ratio (0.88 versus 0.80; **p = 0.002) in DIO mice (Figure 1E). Using a direct RNA hybridization gene expression assay for oligodendrocyte stages, we find that DIO drives an immature OPC-like gene expression profile in white matter compared with control (Figures S2C and S2D; Table S1), suggesting that DIO may compromise myelination by lineage restriction of OPCs.

### DIO impairs post-stroke remyelination

**Figure 2.**
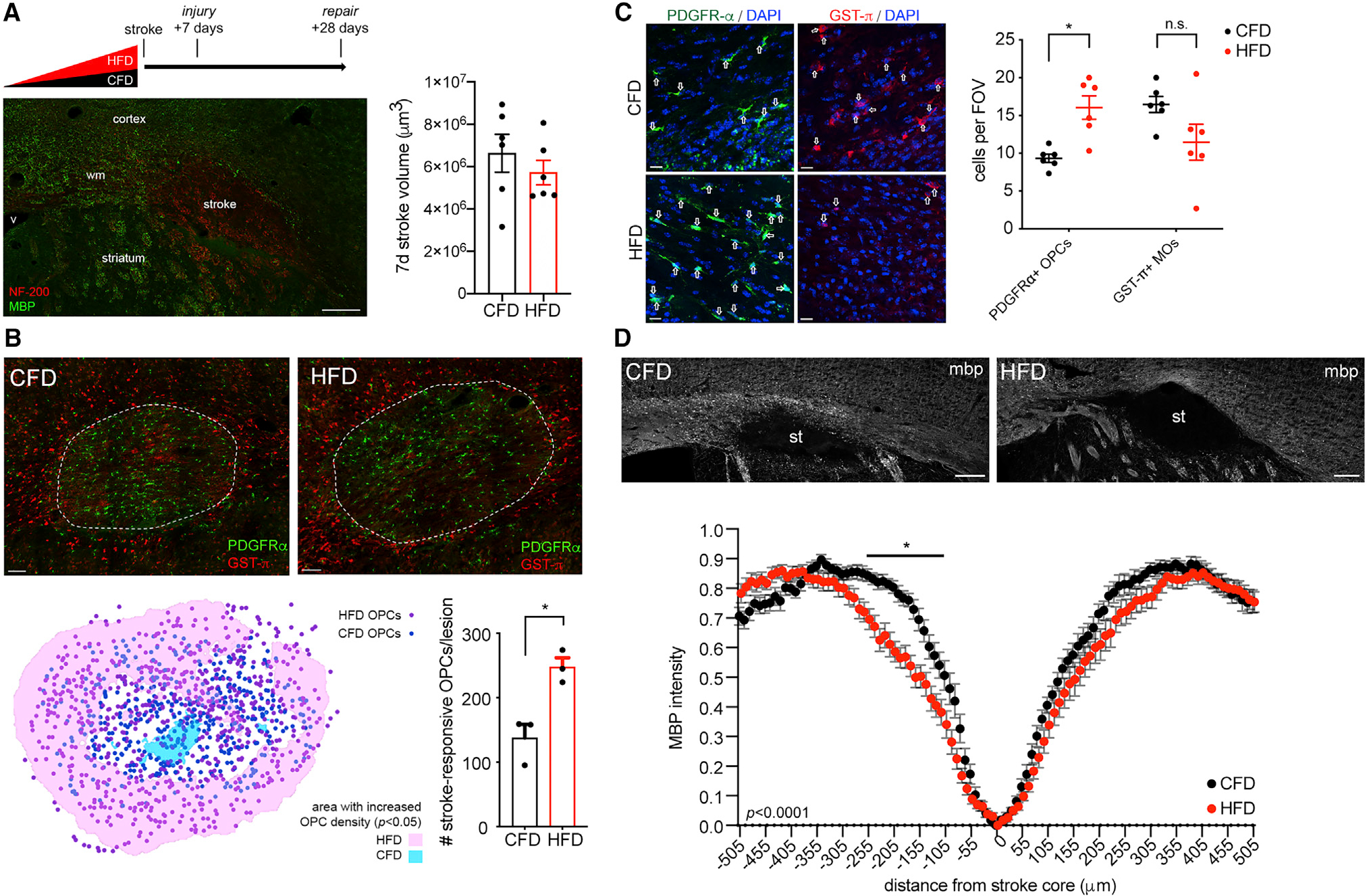
DIO-induced changes in stroke-responsive OPCs and repair after focal white matter stroke Schematic of stroke modeling in DIO (top panel) with representative white matter ischemic lesion shown with labeling for myelin basic protein (MBP; green) and neurofilament-200 (NF-200; red) (left). Graph of white matter stroke volume 7 days post-stroke between animals on CFD versus HFD (6.6 × 10^6^± 604.0 versus 5.7 × 10^6^ ± 485.8 μm^3^, p = 0.31 by Mann-Whitney, n = 6/grp) (right) (A). Labeling of stroke-responsive PDGFRα+ OPCs (green) and GST-π (red) mature oligodendrocytes at 7 days post-stroke (top panels). Spatial mapping of stroke-responsive OPCs in CFD (dark blue) and HFD (purple) with shaded areas indicating regions of stroke lesion with statistically increased stroke-responsive OPCs between CFD (light blue) and HFD (pink) (bottom left panel). Graph of total #OPCs/lesion (*p = 0.013; n = 3/grp) (B). PDGFRα+ OPCs (green, left) and GST-π mature oligodendrocytes (red, right) from stroke lesions at 28 days post-stroke. Graph of oligodendrocyte cell numbers at 28 days post-stroke (*p = 0.0114; n = 5/grp) (C). Myelin basic protein in stroke lesions at 28 days post-stroke (p < 0.0001, *F* = 3.11 by two-way ANOVA; *adjusted p < 0.05 for specific peri-infarct tissue segments) (D). Error bars represent S.E.M. Scale bars: 100 μm (A and D) and 10 μm (B and C).

The major pathologic consequence of advanced cerebral small vessel disease is subcortical ischemic injury to the white matter. To determine the effect of DIO on ischemic white matter injury, we used an established model of white matter stroke produced by focal stereotactic injection of an eNOS inhibitor producing a permanent focal region of ischemia.^30,33^ At 7 days after white matter stroke, there was no significant difference in the stroke lesion volume when comparing animals on CFD versus HFD (p = 0.31) (Figure 2A). This ischemic white matter lesion results in a distinct population of stroke-responsive PDGFRα+ OPCs.^31,34^ In DIO mice, PDGFRα+ stroke-responsive OPCs per lesion were increased compared with control at 7 days post-stroke (Figure 2B). Spatial mapping of stroke-responsive OPCs coupled with nearest neighbor comparative analysis indicates a greater distribution of stroke-responsive OPCs specifically at the peri-infarct lesion margins in DIO mice compared with control (Figures 2B and S3). To determine if DIO impairs OPC differentiation after stroke, we compared PDGFRα+ OPC and GST-π+ mature oligodendrocyte cell counts in three regions of interest spanning the ischemic white matter lesion at 28 days post-stroke. DIO drives a significant change in oligodendrocyte cell populations 28 days after stroke (p = 0.0011, two-way ANOVA, *F* = 14.47) (Figure 2C). Residual stroke-responsive PDGFRα+ OPCs were present at 28 days post-stroke in animals on HFD compared with those on CFD (adjusted p = 0.0114). The number of GST-π+ mature oligodendrocytes within the lesion at 28 days post-stroke was variable and generally reduced in animals on HFD compared with those on CFD (adjusted p = 0.0654). To further assess the effect of DIO on white matter stroke remyelination, we measured peri-infarct myelin basic protein as a function of distance from the stroke core. This measure of functional remyelination after stroke demonstrates reduced peri-infarct MBP+ immunoreactivity at 28 days post-stroke in DIO mice compared with control mice (p < 0.0001, two-way ANOVA, *F* = 3.11) (Figure 2D), indicating a failure of post-stroke remyelination.

### Molecular profiling of white matter endothelia and OPCs using RiboTAG

**Figure 3.**
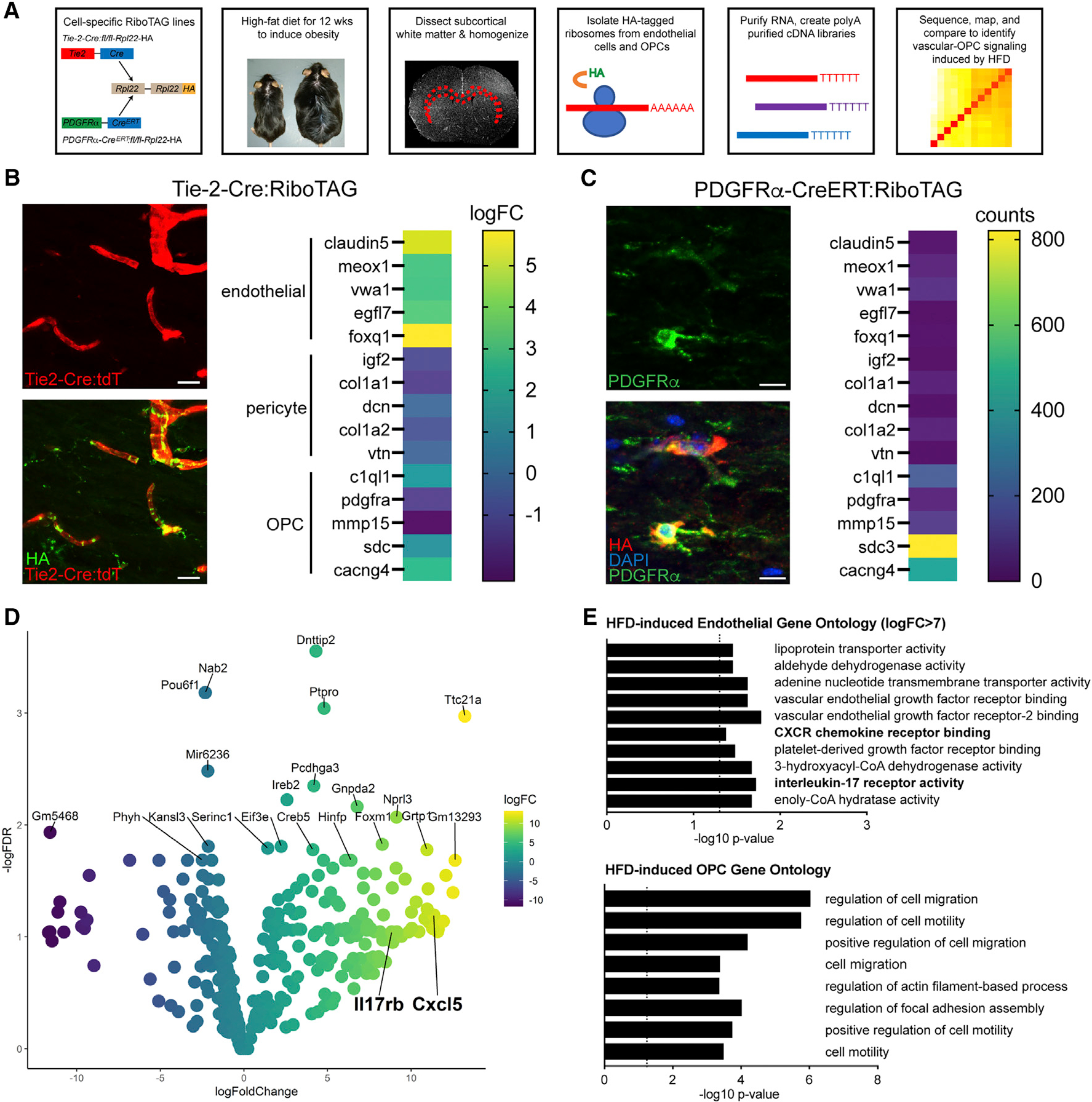
RiboTAG profiling of white matter endothelia and OPCs Schematic representation of workflow for molecular profiling of endothelia and OPCs in chronically injured white matter using translating ribosome affinity purification after DIO (A). Tie2-Cre;tdTomato;RiboTag transgenic mice labeled for HA (green, bottom left panel) enrich for endothelial marker genes (log fold change [FC]) by TRAP-seq (p = 0.0005, *F* = 15.15 by one-way ANOVA; B). PDGFRα-Cre^ERT^;RiboTAG transgenic mice labeled for HA (red, bottom left panel) enrich for OPC marker genes (counts) (p = 0.039, *F* = 4.31 by one-way ANOVA; C). Volcano plot of the top differentially expressed genes (FDR < 0.1) between anti-HA pull-downs from Tie2-Cre;tdTomato;RiboTag mice in CFD and HFD animals (n = 3/grp) (D). Gene Ontology of top up-regulated EndoRiboTAG genes (logFC > 7; FDR < 0.1) (top) and OPC-RiboTAG genes (FDR < 0.1) (bottom) (E). Scale bars: 10 μm. Complete gene list available in Supplemental Tables 2 and 3.

To identify the molecular pathways induced by DIO that could drive abnormal endothelial-OPC signaling and thereby impair baseline and post-stroke remyelination, we used a cell-specific RiboTAG approach employing Tie2-Cre:RiboTag and PDGFRα-CreERT2:RiboTag mice to tag ribosomes in endothelia and OPCs, respectively, enabling translating ribosome affinity purification (TRAP)^35^ (Figure 3A). Tie2-Cre:RiboTag mice show robust hemagglutinin (HA) labeling in the cerebrovasculature (Figure 3B). RNA sequencing (RNA-seq) analysis of immunoprecipitated HA+ ribosomes from Tie2-Cre: RiboTag mice show endothelial specificity, with a specific enrichment of endothelial transcripts compared with established marker genes for other perivascular cells including pericytes and OPCs.^36^ Similarly, PDGFRα-CreERT2:RiboTag mice show significant HA expression in OPCs 4 days after induction with tamoxifen, and OPC transcripts are enriched after TRAP-seq (Figure 3C). In both RiboTag strains, DIO results in a specific gene expression profile (Figures 3D and S4, Tables S2 and S3). Compared with white matter endothelial cells from normal-weight mice, DIO induced 112 up-regulated genes and 60 down-regulated genes (false discovery rate [FDR] < 0.1). Gene Ontology of the up-regulated endothelial genes points to DIO enrichment of immune signaling pathways including C-X-C chemokine signaling and IL receptor activation within white matter endothelia (Figure 3E). Among the top differentially regulated genes, *IL17Rb* (8.83-fold increase, FDR = 0.090) and its effector chemokine *Cxcl5* (11.35-fold increase, FDR = 0.064) were strongly up-regulated genes when comparing DIO versus control animals (Figure 3D) and suggests a cognate inflammatory signaling pathway specific to DIO in white matter endothelia. Furthermore, with the known role of chemokine receptor (CXCR) signaling on OPC migration,^23^ we reasoned that endothelial up-regulation of an IL-17Rb/CXCL5 signaling cascade in DIO mice may function to promote OPC migration to the vasculature. Gene Ontology of the differentially expressed genes (DEGs) induced in HA+ OPCs from PDGFRα-CreERT2:RiboTag mice on HFD compared with the full murine genome enriched for multiple pathways involved in cell migration (Figure 3E). Pathway analysis of DEGs in HFD HA+ OPCs demonstrated enrichment for downstream chemokine signaling with 31 of 198 chemokine signaling pathway genes differentially expressed in HFD HA+ OPCs (FDR = 1.62 × 10^−55^)^37^ (Table 1).

### IL-17Rb and CXCL5 up-regulation in injured white matter vasculature

**Figure 4.**
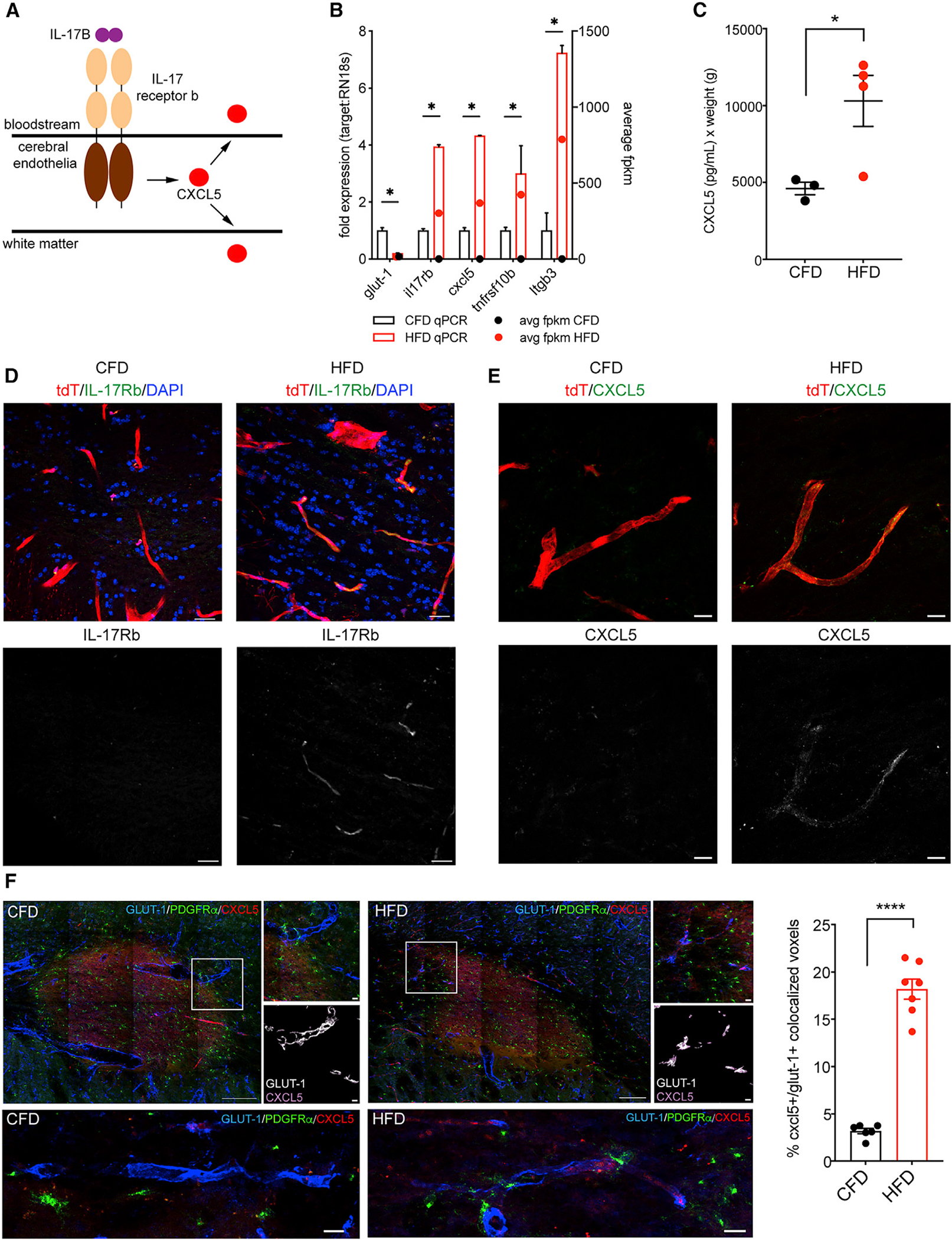
IL-17Rb and CXCL5 up-regulation in injured white matter vasculature Schematic representation of IL-17/CXCL5 signaling in chronically injured cerebral endothelia (A). TRAP-qPCR fold expression compared with average fpkm of top DEGs from white matter endothelia (*adjusted p < 0.05) (B). Weight-adjusted ELISA values (pg/mL) for murine CXCL5 in retro-orbital blood samples from CFD (black) and HFD (red) animals (n = 4/grp, p = 0.0355) (C). Immunofluorescence labeling for IL-17Rb (green, D) and CXCL5 (green, E) is absent in white matter vasculature of Tie2-Cre;tdTomato mice on CFD (left panels) and abundant in white matter vasculature of Tie2-Cre;tdTomato mice on HFD (right panels). Single-channel labeling for IL17Rb (bottom panels, D) and CXCL5 (bottom panels, E) show heterogeneous endothelial expression. Labeling for GLUT-1 (blue), CXCL5 (red), and PDGFRα (green) at 7 days post-stroke in animals on CFD (left) and HFD (right). Inset boxes from the peri-infarct tissue (top) masked for GLUT-1 (white) with only co-localized CXCL5 (purple) (bottom). Graph of percentage of co-localized CXCL5+/GLUT-1+ voxels (****p < 0.0001) (F). Error bars represent S.E.M. Scale bars: 50 μm (F), 20 μm (D), and 10 μm (E).

IL-17 signaling involves five IL ligands (A–E) and five cognate receptor isoforms that hetero- and/or homo-dimerize to effect downstream signaling.^38^ Within our transcriptional dataset, the only IL-17 receptor isoform that was significantly differentially regulated in DIO-affected cerebral endothelial cells was IL-17Rb (Table S4). Among a number of diverse functions, IL-17 receptor activation drives effector chemokine signaling, including CXCL5^39^ as a mechanism of identifying tissue injury. CXCL5 is a member of the C-X-C chemokine family^40^ that acts as a chemoattractant in other tissues and has been reportedly up-regulated in white matter after peri-natal hypoxia.^41^ Guided by our RNA-seq data, we hypothesized that DIO may induce IL-17B signaling acting through IL-17Rb resulting in increased endothelial expression of CXCL5, resulting in its secretion both into the bloodstream and into surrounding brain tissue to exert a localized paracrine effect on OPCs (Figure 4A). First, to confirm DIO-induced up-regulation of IL-17Rb/CXCL5 in white matter endothelia observed by TRAP-seq, we performed TRAP-qPCR using independent Tie2-Cre:RiboTag biologic replicates for a subset of differentially regulated genes (*Glut-1*, *Itgb3*, *Cd180*, *Hsd3b3*, *Tnfrsf10b*, *Il17rb*, *Cxcl5*, and *Ttc21a*) (Figure 4B). Using TRAP-qPCR, we confirmed the effect of DIO on white matter endothelia with similar degrees of up-regulation for *Il17rb* and *Cxcl5* (3.94 ± 0.07-fold expression, p = 0.0009, and 4.32 ± 0.01-fold expression, p = 0.0009, respectively). Retro-orbital venous blood sampling confirmed increased serum detection of CXCL5 in DIO mice (4,609 ± 407 versus 10,306 ± 1,660 pg/mL, p = 0.036; Figure 4C). Immunofluorescent labeling for IL-17Rb (Figure 4D) and CXCL5 (Figure 4E) in Tie2-Cre;tdTomato (Ai14) mice demonstrated a marked increase in detection of both molecules within white matter cerebral vessels in DIO mice. In peri-infarct tissue 7 days after subcortical white matter stroke, endothelial CXCL5 expression is significantly increased in mice on HFD versus those on CFD as measured by the percentage of CXCL5+ voxels that co-localized with GLUT-1 within the peri-infarct tissue surrounding the stroke (Figure 4F). As in uninjured white matter, the percentage of CXCL5+/GLUT-1+ voxels was significantly increased within the periinfarct tissue in animals on HFD (3.18 ± 0.29 versus 18.19 ± 1.06; p < 0.0001). Importantly, OPCs were seen in close apposition to CXCL5+ vessel segments in DIO mice, suggesting that this IL-chemokine cascade may drive vascular-OPC signaling and regulate OPC migration (Figure 4F).

### The IL-17/CXCL5 pathway as a vessel-to-OPC signaling paradigm

**Figure 5.**
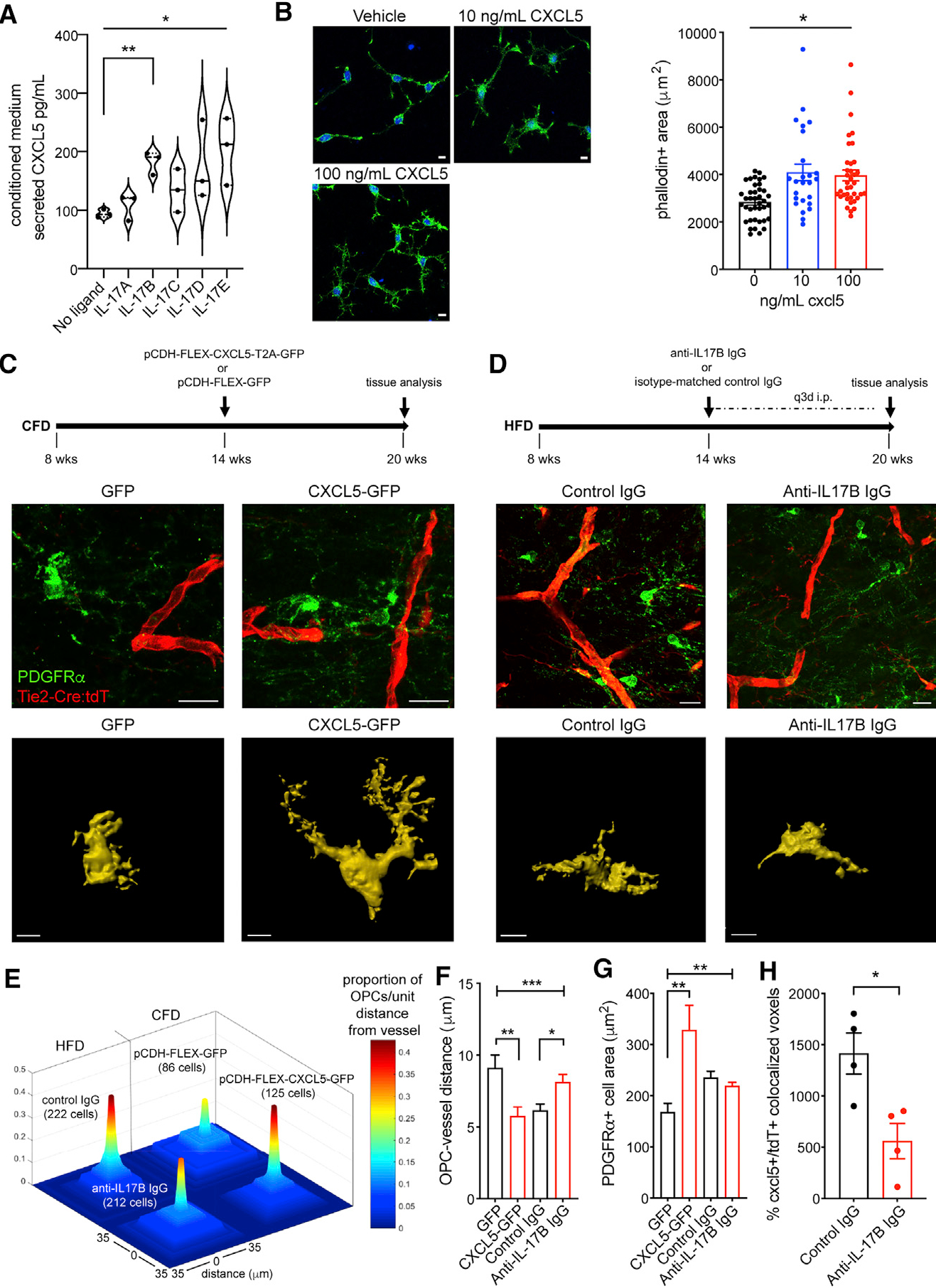
IL-17B/IL-17Rb/CXCL5 signaling is a vessel-to-OPC signal in white matter vasculature Human brain microvascular endothelial cells were stimulated with IL-17 ligands A–E (250 ng/mL) and CXCL5 levels measured in conditioned media 48 h after stimulation (*p = 0.0372 by Kruskal-Wallis H test; **post-hoc comparison for IL-17B versus no ligand, adjusted p = 0.0178) (A). Phalloidin+ cellular area in O4+ OPCs grown *in vitro* exposed to vehicle (top panel) or recombinant CXCL5 (bottom panel) for 48 h (p < 0.0001, *F* = 9.82 by one-way ANOVA) (B). Approach for CXCL5 transgenic-viral gain of function in subcortical white matter of Tie2-Cre;tdTomato mice (top panel) (C). PDGFRα+ OPC (green) labeling in GFP-transduced Tie2-Cre;tdTomato mice (red, left panel) and CXCL5-GFP-transduced Tie2-Cre;tdTomato mice (right panel). Representative masked cellular profiles of PDGFRα+ cell area (bottom panels). Schematic of anti-IL-17B antibody treatment (top panel) (D). PDGFRα+ OPC (green) labeling in control IgG-treated Tie2-Cre:tdT mice (left panel) and anti-IL-17B IgG-treated Tie2-Cre:tdT mice (right panel). Representative masked cellular profiles of PDGFRα+ cell area (bottom panels). Proportion of OPCs per unit distance from vessel (0–35 μm) in each condition (total measured cell number per condition in parentheses) (E). Average distance of OPCs to vessel (***p = 0.0005, *F* = 6.06 by one-way ANOVA; **adjusted p = 0.0039; *adjusted p = 0.0168) (F). Average *in vivo* PDGFRα+ OPC cell area (**p = 0.0068, *F* = 7.38 by one-way ANOVA; **adjusted p = 0.002) (G). Graph of co-localized CXCL5+/GLUT-1+ voxels in anti-IL-17B IgG-treated animals (n = 4/grp; *p = 0.018) (H). Error bars represent S.E.M. Scale bars: 10 μm

To confirm that IL-17 signaling can drive brain endothelial CXCL5 secretion as suggested by our transcriptional data and working model, we stimulated primary human brain microvascular endothelial cells with recombinant isoforms of IL-17 (A–E). IL-17B, -D, and -E (250 ng/mL) were noted to drive 2-fold increases in the secretion of CXCL5 into conditioned medium (p = 0.0372; Figure 5A). *In vitro* exposure of O4+ OPCs to increasing doses of recombinant murine CXCL5 resulted in a dose-dependent increase in OPC cell area with cytoskeletal changes suggesting motility (p < 0.0001, *F* = 9.82 by one-way ANOVA; Figure 5B). To determine the ability of endothelial CXCL5 to signal to OPCs *in vivo*, we used a combined transgenic and targeted viral gene expression approach (Figure 5C). We designed a pCDH-FLEX-CXCL5-T2A-GFP lentiviral construct to target CXCL5 overexpression to white matter endothelial cells in Tie2-Cre;tdTomato mice. Injection of lentiviral particles expressing either pCDH-FLEX-CXCL5-T2A-GFP or control pCDH-FLEX-GFP into the subcortical white matter of Tie2-Cre;tdTomato mice results in targeted gene expression specifically in white matter vasculature (Figure S5). After 6 weeks of endothelial upregulation of CXCL5-GFP or GFP in normal-weight mice, we measured the distance of individual OPCs from vessels and the cell area of vessel-associated OPCs (Figure 5C). The average distance of OPCs from tdT+ vessels was reduced in CXCL5-GFP-injected animals compared with GFP-injected animals, while the number of PDGFRα+ OPCs in apposition to tdT+ vessels was increased (top panels in Figures 5C, 5E, and 5F), supporting a chemoattractant role for CXCL5 on OPCs. Consistent with the effects of recombinant CXCL5 on OPCs *in vitro*, endothelial over-expression of CXCL5 *in vivo* resulted in increased OPC cell area (bottom panels in Figures 5C and 5G). There was no difference in vessel length induced by CXCL5 overexpression in normal-weight mice (0.26 ± 0.05 mm [GFP] versus 0.25 ± 0.06 mm [CXCL5-GFP]; p = 0.89).

To block DIO-induced endothelial CXCL5 expression resulting from IL-17Rb activation, we employed repetitive peripheral injections of a function-blocking anti-IL-17B antibody or isotype control immunoglobulin G (IgG) for 6 weeks in Tie2-Cre;tdTomato mice on HFD (Figure 5D). Endothelial CXCL5 expression within the tdT+ vasculature of subcortical white matter was reduced by 60.4% using this approach (p = 0.018, n = 4/group [grp]; Figure 5H), while IL-17Rb levels were not changed (Figure S5), indicating that DIO-induced increases in endothelial CXCL5 can be at least partially regulated through IL-17B signaling at the endothelial cell surface. Peripheral blocking of IL-17B signaling significantly reduced both the frequency of vessel-associated OPCs as well as the mean vessel-OPC distance in DIO mice (top panels in Figures 5D–5F), while the cell surface area of vessel-associated OPCs was not significantly different in HFD mice administered anti-IL-17B antibody (bottom panels in Figures 5D and 5G). Notably, anti-IL-17B IgG treatment did not significantly alter white matter vessel length in mice on HFD (0.22 ± 0.04 mm [control IgG] versus 0.21 ± 0.04 [anti-IL-17B IgG]; p = 0.50).

### IL-17B and CXCL5 levels in human subjects at risk for cerebrovascular disease

**Figure 6.**
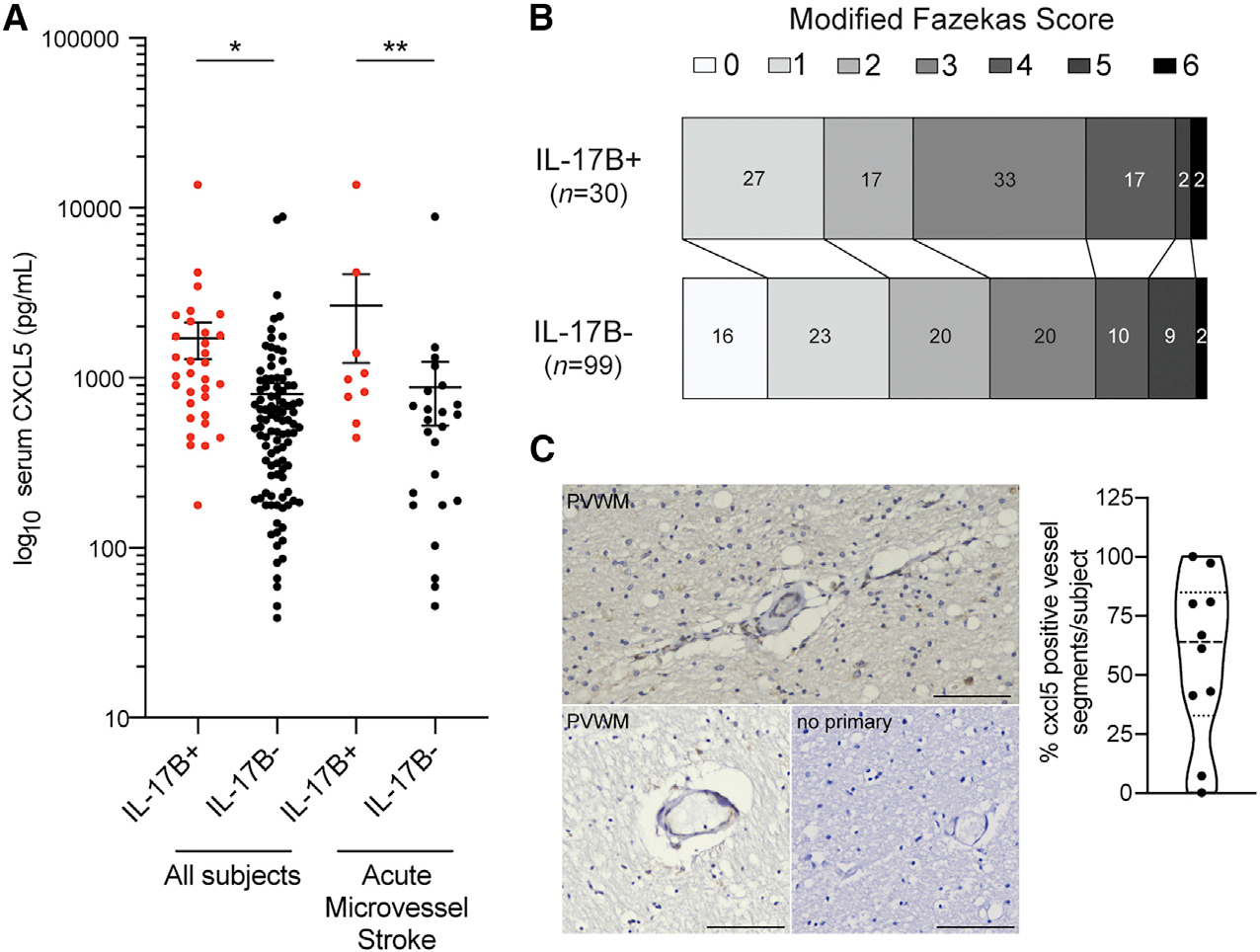
CXCL5 as a biomarker of cerebral small vessel disease Plasma levels of log_10_-CXCL5 in ASPIRE cohort subjects separated by detectable plasma IL-17B (n = 32; median 1043.0 pg/mL) compared with those with undetectable plasma IL-17B (n = 99; median 515.3 pg/mL; *p < 0.0001). Plasma log_10_-CXCL5 levels in subjects with MRI-confirmed acute microvascular ischemia (IL-17B + subjects; n = 9; 978.2 pg/mL versus IL-17B− subjects; n = 24; 539.7 pg/mL) (**p = 0.0157) (A). Ordinal shift analysis of modified Fazekas scale scores from plasma IL-17B+ and IL-17B− subjects (p < 0.0001) (B). Representative immunohistochemical detection of CXCL5 in human frontal white matter vasculature in subjects with cerebrovascular pathology (C). Percentage of CXCL5+ vessel segments in peri-ventricular white matter (n = 10) (p = 0.0005). Error bars represent S.E.M. Scale bar: 10 μm

With a working model suggesting that DIO drives white matter endothelial CXCL5 expression through IL-17B/IL-17Rb signaling, we sought to establish the relevance of this signaling cascade to human cerebral small vessel disease and vascular cognitive impairment. Using available plasma samples from a single-center cohort study including subjects presenting with acute neurologic symptoms suggestive of stroke,^42,43^ we assayed plasma levels of IL-17B and CXCL5 using a custom Luminex assay. In those subjects with concurrent blood samples and MRI scans (n = 131), subjects with detectable levels of IL-17B (n = 32, mean IL-17B = 47.83 pg/mL) had higher median CXCL5 levels (1,043.0 pg/mL) than in those without detectable IL-17B (n = 99, 515.3 pg/mL; p < 0.0001) (Figure 6A). In subjects with tissue-confirmed acute microvessel ischemic lesions, CXCL5 values were higher in those subjects with detectable IL-17B compared with those without measurable IL-17B levels (p = 0.0157) (Figure 6B). In this cohort, the burden of pre-existing cerebral small vessel disease indicated by modified Fazekas scale scoring of white matter hyperintensities is significantly different in IL-17B+ subjects compared with IL-17B− subjects (p < 0.0001). To confirm CXCL5 expression by white matter endothelia, we examined CXCL5 expression in peri-ventricular white matter from a small post-mortem convenience cohort (n = 10) of older individuals (86 ± 8 years of age) with measurable amounts of cerebrovascular pathology (Table S5; Figure 6C). The mean percentage of CXCL5+ vessel segments per subject was 71.2% ± 0.08% (17.2 ± 3.4 vessel segments/subject; p = 0.0005) (Figure 6C). Using a separate cohort of 150 subjects with baseline serum sampling and longitudinal cognitive assessment, a mixed-effects regression model adjusted for age, sex, education, and pre-morbid cognitive diagnosis indicates that elevated serum CXCL5 values are significantly associated with level of decline in mean executive function over time (β estimate = 4.61 × 10^−5^, p = 0.026) (Table S6).

## DISCUSSION

Cerebral small vessel disease is increasingly recognized as a substantial contributor to stroke risk and dementia.^6^ Microvascular injury in the brain is driven by cardiovascular risk factors, yet molecular factors that link systemic vascular risk factors with molecular pathways in the brain are lacking. Here, we use a mouse model of DIO to identify a multicellular inflammatory signaling cascade active in injured white matter before and after ischemic stroke that can also function as a diagnostic and prognostic biomarker for cerebral small vessel disease. Modeling of chronic cerebrovascular risk and pathology using a combined DIO and subcortical white matter stroke model demonstrate that OPCs respond to DIO by vascular association and that their differentiation post-stroke is restricted. Using TRAP-seq in endothelial and OPC transgenic mice, we identify a vascular-OPC signaling cascade acting predominantly through IL-17B-IL-17Rb interaction at the vascular surface to drive endothelial expression of the C-X-C family chemokine CXCL5, promoting OPC chemoattraction to the vasculature. With a combination of *in vitro* and *in vivo* studies, we show both that IL-17B regulates endothelial expression of CXCL5 and that OPCs respond to endothelial CXCL5 expression by associating to the vasculature, likely through CXCR-mediated activation of cellular migration. Finally, we extend these findings to the human condition by demonstrating that CXCL5 is present in aged cerebral small vessels and that circulating levels of CXCL5 can identify subjects with imaging or cognitive manifestations of cerebral small vessel disease.

Despite advances in single-cell RNA-seq, cell-specific transcriptional profiling using ribosomal tagging remains a valuable tool in parsing out molecular signals from a complex tissue such as the brain.^35,44^ Here, we utilized EndoRiboTag mice^45^ in the context of a chronic vascular risk factor model to identify endothelial pathways that appear relevant to human cerebral small vessel disease. A similar vascular profiling approach could be easily applied to identify microvascular injury signals in other organs such as the kidney or retina or conditions that feature microvascular injury including aging, diabetes, or isolated hypertension. Our attempt to translate this vascular profiling approach from mouse to human as a platform for biomarker discovery may represent a unique opportunity to better understand the relationship between cerebrovascular risk factors and human cerebral small vessel disease.

Here, we chose to model obesity as it is a leading cardiovascular and cerebrovascular risk factor, is growing in prevalence,^46^ is associated with white matter changes in humans,^11,13–15^ and has a reliable animal model.^28^ Our findings of reductions of white matter vasculature and increased OPCs in DIO mice are similar to those reported in other models of chronic white matter injury.^26^ While these results reporting vessel-associated OPC morphology in the context of DIO are somewhat discrepant with the heterogeneity of OPC morphologies reported by others,^21^ this work focused exclusively on subcortical white matter and peri-infarct OPCs, which may have less variation in OPC morphology to begin with. Our results showing ultrastructural changes in myelin in adult-onset DIO are similar to those seen in genetically obese (ob/ob) mice with reductions in myelin^47^ and increases in OPCs in leptin-deficient ob/ob mice,^48^ validating this model for the study of chronic white matter injury. OPCs are known to respond early and robustly to white matter ischemic lesions common to the aging human brain.^31,49,50^ The peri-infarct white matter at the margin of the ischemic lesion, often referred to as the white matter penumbral region,^51^ is where reparative remyelination can be activated.^31^ In DIO mice, the stroke-responsive OPC lesion area is 30% larger, and this expanded penumbral region is marked by increased endothelial CXCL5 expression, potentially explaining why more stroke-responsive OPCs are seen at the lesion periphery. Though we did not demonstrate it here, therapeutic targeting of the vasculature in order to regulate remyelination after stroke is an attractive strategy for brain repair.

Vessels and OPCs are known to interact both during development and to maintain white matter homeostasis.^52^ During CNS development, OPCs migrate extensively to distribute throughout the entire CNS, and this migration requires the physical vascular scaffold.^23^ Cerebral endothelial cells secrete trophic factors that activate Src and Akt signaling pathways to support the survival and proliferation of OPCs.^18^ However, the full spectrum of molecular pathways that drive the vessel-OPC interaction remain largely unknown. The present data in disease and studies in the developing brain indicate that chemokines are critical. *In vivo* time-lapse imaging reveals that in the developing mouse brain, OPCs interact with vasculature and migrate along the vessels to the destined cerebral regions dependent on CXCR4 activation in OPCs, which binds to endothelial secreted ligand CXCL12, and promotes their attraction to cerebral vasculature.^53^ Our study illustrates a similar phenomenon, with DIO-induced endothelial expression of CXCL5 promoting the association of OPCs to the vasculature within adult white matter *in vivo*. Transcriptional profiling of OPCs in DIO using PDGFRα RiboTAG mice further imply that chemokine signaling pathways play a significant role in regulating a migratory interaction between endothelial cells (ECs) and OPCs. Based on the Gene Ontology analysis from DIO OPCs, this interaction may promote white matter angiogenesis in the chronic state.

Though much is known about the IL-17 superfamily, comparatively little is known about IL-17B and IL-17Rb signaling.^38^ Using both gain- and loss-of-function studies *in vitro* and *in vivo*, we clearly demonstrate that IL-17B can act on brain endothelia to produce CXCL5. The precise source of IL-17B is unclear, though DIO is known to promote Th17 T cells that may function as a primary source of this cytokine.^54^ Beyond its potential paracrine action on OPCs in the white matter, CXCL5 is secreted by ECs. As such, we hypothesized that circulating CXCL5 could also function as a disease biomarker. In two small, but independent, cohort studies, we show that circulating CXCL5 can function as a cross-sectional diagnostic biomarker for white matter injury on MRI and, in a longitudinal cohort, may associate with future cognitive impairment.

An emerging concept places the cerebral EC at the center of the pathophysiology relevant to cerebral small vessel disease.^55^ Because they act as the conduit between the brain and systemic insults such as hypertension, diabetes, and the metabolic disturbances of obesity, the cerebral endothelia represent an attractive target for understanding disease pathogenesis. From the data presented here, intercellular inflammatory signaling involving the IL-chemokine pathway may be central to white matter injury and post-ischemic myelin repair. Molecular pathways triggered by chronic cerebrovascular risk factors can directly alter injury response and repair after stroke by acting through vascular regulation of myelination.

### Limitations of the study

Despite our translational results from mouse to human, this study has important limitations. Regional transcriptional profiling from endothelia only in the subcortical white matter limits the ability to generalize this oligovascular signaling response to other brain regions. Additionally, while we demonstrate the ability of IL-17B to signal through the IL-17Rb receptor to trigger CXCL5 expression in murine and human microvascular ECs, we did not identify a source for circulating IL-17B. If identified, this could drive a therapeutic strategy for white matter repair by targeting the source of IL-17B in obesity. Finally, both cohorts of human subjects are relatively small, and though significant, the magnitude of the effect on diagnosis or prognosis is small. Future studies can expand on these findings using combined IL-17B and CXCL5 measurements in larger, coordinated cohorts enriched for subjects at risk for vascular cognitive impairment.

## STAR★METHODS

### RESOURCE AVAILABILITY

#### Lead contact

Further information and requests for resources and reagents should be directed to and will be fulfilled by the lead contact, Jason D. Hinman (jhinman@mednet.ucla.edu).

#### Materials availability

Plasmids generated in this study are deposited in Addgene. Mouse lines generated in this study are available to share upon contact with the lead contact. Anti-IL-17B antibody used in Luminex assay may be available from the manufacturer upon request (Biotechne).

#### Data and code availability

RNA-seq data have been deposited at GEO: GSE217356 and are publicly available as of the date of publication. Accession numbers are listed in the key resources table. Microscopy data reported in this paper will be shared by the lead contact upon request. All original code has been deposited at https://doi.org/10.17605/OSF.IO/2YMB4 and is publicly available as of the date of publication. Any additional information required to reanalyze the data reported in this paper is available from the lead contact upon request.

### EXPERIMENTAL MODEL AND SUBJECT DETAILS

#### Animals

All animal studies presented here were approved by the UCLA Animal Research Committee ARC#2014-067-01B, accredited by the AAALAC. Mice were housed under UCLA regulation with a 12-hour dark-light cycle. All mice used in the study were male. Wild-type C57Bl/6 mice fed ad lib on 60%kCal from fat chow (Research Diets, Inc.) (HFD) (Strain #380050) or 10%kCal from fat chow (Research Diets, Inc.) (CFD) (Strain #380056) were purchased directly from Jackson Labs at 17 weeks of age and allowed to acclimate for 2 weeks prior to experimental use. Weights (g) were measured weekly. The PDGFRα- CreERT2/Rpl22-HA and Tie2-Cre/Rpl22-HA transgenic strain were generated by crossing PDGFRα- CreERT2 mice (Jackson Labs Strain #018280 - B6N.Cg-Tg(Pdgfra-cre/ERT)467Dbe/J) and Tie2-Cre (Jackson Labs Strain #008863-B6.Cg-Tg(Tek-cre)1Ywa/J) with Rpl22-fl-Rpl22-HA (Jackson Labs Strain #011029 - B6N.129-Rpl22tm1.1Psam/J). The Tie2-Cre;tdTomato mice were generated by crossing Tie2-Cre mice with flox-stop tdTomato mice (Jackson Labs Strain #007908 – B6;129S6-Gt(ROSA)26Sortm14(CAG-tdTomato)Hze/J). Diet-induced obesity was induced in transgenic mice by ad lib feeding with 60%kCal from fat chow (HFD) or 10%kCal from fat chow (CFD) (Research Diets, Inc.). Genotyping was performed by transgene specific qPCR (Transnetyx). For OPC RNA-sequencing, tamoxifen (Sigma) was dissolved in corn oil and injected i.p. (50mg/kg) once to PDGFRα-CreERT2/Rpl22-HA (n = 6) mice and animals were euthanized and tissue collected 48 hrs later for RiboTag pulldown as described.

#### Human subjects

##### ASPIRE study cohort

Patients presenting for emergency evaluation of stroke or cerebrovascular disease were recruited and provided blood samples and neuroimaging data as approved by the UCLA Institutional Review Board (IRB # 14-001798) as previously reported.^42^ Serum levels of IL-17B and CXCL5 were measured in technical duplicate using a custom Luminex assay (R&D Systems). Manufacturer protocol was followed and antigen binding within the assay was measured on a Luminex 200 System and analyzed using Milliplex Analyst 5.1. Modified Fazekas scores were determined by blinded analysis of T_2_-weighted FLAIR images by two independent reviewers. ASPIRE study data are available at https://osf.io/92erq/.

##### Post-mortem cohort

Subjects were selected from a subset of 950 UCDavis ADC Neuropathology Core samples based on a priori selection criteria. All subjects consented to autopsy. A convenience cohort of ten elderly individuals with variable amounts of cerebrovascular disease and low Braak and Braak scores were selected for analysis. Age and sex information is provided in Table S2.

##### Longitudinal cohort

UCD ADRC Longitudinal Diversity Cohort consists of demographically diverse individuals recruited through both clinical and community sources.^56^ Formal written consent was obtained for all participants prior to the collection of data. For this study, this highly demographically diverse cohort consists of 58% non-Hispanic Caucasians (Whites), 19% African Americans (Blacks) and 13% Hispanics, 52% female, average age 78 + 7.3 years with average educational attainment of 14.7 + 4.0 years ranging from 0–20 years and various medical comorbidities common to the general population. Longitudinal cognitive testing utilized the Spanish English Neuropsychological Assessment Scale.^57,58^ Participants for this study were assessed 6.3 + 3.6 times ranging from 1–17 times. Serum levels of CXCL5 were measured in technical duplicate using a custom Luminex assay as above.

#### Human brain microvascular endothelial cell culture

Primary Human Brain Microvascular Endothelial Cells (HBMECs) (Cell Systems) between P5-P9 were maintained at 37°C until confluence with manufacturer recommended media containing serum with media exchange every two days. Maintenance cultures were replated into a 96-well filter bottom plate and cultured until near confluence. Cultures were mixed sex and not authenticated.

### METHOD DETAILS

#### Animals

All animal studies presented here were approved by the UCLA Animal Research Committee, accredited by the AAALAC. Mice were housed under UCLA regulation with a 12 hour dark-light cycle. All mice used in the study were male. Wild-type C57Bl/6 mice fed ad lib on 60%kCal from fat chow (HFD) (Strain #380050) or 10%kCal from fat chow (CFD) (Strain #380056) were purchased directly from Jackson Labs at 17 weeks of age and allowed to acclimate for 2 weeks prior to experimental use. The PDGFRα- CreERT_2_/Rpl22-HA and Tie2-Cre/Rpl22-HA transgenic strain were generated by crossing PDGFRα- CreERT_2_ mice (Jackson Labs Strain #018280 - B6N.Cg-Tg(Pdgfra-cre/ERT)467Dbe/J) and Tie2-Cre (Jackson Labs Strain #008863-B6.Cg-Tg(Tek-cre)1Ywa/J) with Rpl22-flRpl22-HA (Jackson Labs Strain #011029 - B6N.129-Rpl22tm1.1Psam/J). The Tie2-Cre;tdTomato mice were generated by crossing Tie2-Cre mice with flox-stop tdTomato mice (Jackson Labs Strain #007908 – B6;129S6-Gt(ROSA)26Sor^tm14(CAG-tdTomato)Hze^/J). Diet-induced obesity was induced in transgenic mice by ad lib feeding with 60%kCal from fat chow (HFD) or 10%kCal from fat chow (CFD) (Research Diets, Inc.). Weights (g) were measured weekly. For OPC RNA-sequencing, tamoxifen (Sigma) was dissolved in corn oil and injected i.p. (50mg/kg) once to PDGFRα-CreERT^2^/Rpl22-HA (*n* = 6) mice and animals were euthanized and tissue collected 48 hrs later for RiboTag pulldown as described.

#### White matter stroke

Subcortical white matter ischemic injury was induced as previously described^33^ using three stereotactic injections of the irreversible eNOS inhibitor, L-Nio (L-N⁵-(1-Iminoethyl) ornithine, dihydrochloride; Calbiochem) into the subcortical white matter under sensorimotor cortex. Animals (*n* = 8/grp) were sacrificed at 7- or 28-days post-stroke and analyzed for tissue outcomes.

#### Translating ribosome affinity purification and RNA-sequencing

HA-tagged ribosomal associated RNAs from cerebral white matter endothelia or OPCs were isolated following published protocol.^35^ Post-immunoprecipitation RNA samples were purified by Nucleospin miRNA kit (Machary-Nagel). Normalized RNA amounts (ng) underwent cDNA library generation using the TrueSeq with Ribozero kit preparation (Illumina), pooled and sequenced using 69 bp paired end reads on a Illumina HiSeq 4000 sequencer. Samples were sequenced over 4 lanes for an average of read count of 62.1± 10.7 million per sample (Tie2-Cre:RiboTag) and 75.9 ± 11.1 million per sample (PDGFRα- CreERT_2_:RiboTag). Reads were aligned to the mouse genome using STAR (v.mm10). Differential gene expression analysis was performed using EdgeR assuming an FDR <0.1 as significant. Gene ontology analysis was performed using GOrilla^59^ and Enrichr.^60^ Chemokine pathway analysis was performed using the KEGG pathway resource^61^ and verified using the STRING database resource.^37^ Selected genes were verified by qPCR using independent TRAP isolates.

#### RNA hybridization assay

Wild-type C57Bl/6 mice (*n* = 4/grp) were placed on CFD or HFD starting at 8 weeks of age and after 12 weeks on CFD or HFD were sacrificed. The subcortical white matter was freshly dissected. RNA was isolated using the Nucleospin miRNA kit (Machary-Nagel). RNA samples were allowed to directly hybridize with a custom RNA probe set for 120 oligodendrocyte/myelin gene set derived from Zhang et al.^36^ with 40 genes each corresponding to the major oligodendrocyte stages including oligodendrocyte progenitor cells (OPC), pre-myelinating oligodendrocytes (PMO), and (myelinating oligodendrocytes (MO). Hybridized mRNA species were detected using the nCounter detection system (Nanostring) and normalized to five housekeeping genes (Supplemental Data File 1). Normalized counts for each probe set were divided into three major oligodendrocyte subtypes (OPC, PMO, and MO) and compared by differential gene expression analysis. Additional comparisons were performed using normalized read counts from Zhang et al. using a tSNE data reduction analysis.

#### IL-17 treatment and CXCL5 measurement

Two days after seeding, HBMECs were stimulated with culture medium containing 250 ng/mL of mouse IL-17A, B, C, D, or E (R&D Systems, Inc.). Conditioned media from triplicate culture conditions was collected after 48 hours and human CXCL5 levels measured using a human CXCL5 Quantikine Elisa Kit (R&D Systems, Inc.). Absorbance values measured at 450 nm and absorbance at 570 nm was used for background subtraction. Background subtracted absorbance values were converted to pg/mL concentrations based on standard curve concentrations.

#### Microscopy and imaging

Animals were euthanized with a lethal dose of isoflurane, transcardially perfused with PBS followed by 4% paraformaldehyde in 0.1 M sodium phosphate buffer, brains removed, post-fixed for 24 hrs and cryoprotected for 48 hrs in 30% sucrose in PBS. Forty micron coronal cryosections and immunostaining were performed essentially as described.^30^ The following primary antibodies were used: mouse anti-NF200 (1:200, Sigma), rabbit anti-MBP (1:500, Calbiochem), goat anti-PDGFRα (1:500; Neuromics), mouse anti-HA (1:1000, Biolegend), rabbit-Gst-π (1:1000, Millipore), rabbit anti-IL-17Rb (1:500, Santa Cruz Biotech), rat anti-CXCL5 (1:250, R&D) in PBS containing 5% goat or donkey serum and 0.3% Triton-X 100 (Sigma) overnight at 4°C. Secondary antibody labeling was performed using donkey anti-mouse, donkey anti-rabbit, donkey anti-rat or donkey anti-goat Fab_2_-Alexa conjugated antibodies (Jackson Immunoresearch, Inc.). All microscopic images were obtained using a Nikon C2 confocal microscope.

#### Electron microscopy

Wild-type C57Bl/6 mice (*n* = 6/grp) on CFD or HFD were transcardially perfused with a 2% glutaraldehyde solution, post-fixed for 24 hrs, hemisected in the sagittal plane and 2 mm cubes including the corpus callosum were dissected and embedded in plastic resin for ultrastructural analysis as previously described.^31^ One-micron, plastic embedded toluidine blue stained sections were used to select transcallosal fibers underneath sensorimotor cortex by light microscopy. Three electron micrographs were obtained at a primary magnification of 7200X using a JEOL 100 CX transmission electron microscope and a representative electron micrograph of high technical quality from each animal was used for quantitation of fiber diameter, axon diameter, myelin thickness, and g-ratio.

#### Lentiviral injection

A plasmid containing the open reading frame of the murine CXCL5 sequence with a 3′ stop codon was purchased from Origene (#MR200761). The pCDH-EF1-FLEX-EGFP-CMV-2A-TagBFP2-SC dual promoter lentiviral backbone was created by subcloning the FLEX-GFP sequence between the loxP sites from the pAAV-FLEX-GFP vector (Addgene #28304) into the pCDH-EF1-MCS-CMV-2A-pTagBFP2-SC dual promoter lentiviral construct using restriction digestion. The pCDH-EF1-FLEX-EGFP-CMV-2A-TagBFP2-SC backbone was linearized by removing the GFP sequence between the loxP sites using restriction digestion with XhoI and EcoRI (New England Biolabs). The murine CXCL5 sequence was PCR amplified in a HiFi DNA Assembly reaction (New England Biolabs) such that it was subcloned in the 3’>5′ position in between the loxP sites. The resulting reaction was transformed into Stbl3 E.coli cells and positive clones were identified by restriction digestion and verified by DNA sequencing. Subsequently, a 3’>5′ T2A-copGFP sequence was added 5′ to the murine CXCL5 sequence. The donor T2A-copGFP vector (pCDH-EF1-MCS-copGFP; System Biosciences) was PCR amplified and subcloned into pCR-Blunt II TOPO (ThermoFisher Scientific) for amplification and utilized in a HiFi DNA Assembly reaction. The resulting reaction was transformed as above and positive clones were identified by restriction digestion and DNA sequencing. DNA amplification was performed using an Endotoxin-Free PureLink Plasmid Midiprep Kit (ThermoFisher Scientific). Resulting DNA was quantified and used in lentiviral packaging. Control GFP and CXCL5-GFP lentivirus were packaged in human 293 cells (ATCC cat. no. CRL-11268) and concentrated by ultracentrifugation on a sucrose column. 200 nL of concentrated virus was injected into the subcortical white matter and allowed to express for 6 weeks.

#### Anti-IL-17B antibody administration

Anti-mIL-17B function blocking antibody (R&D, AF1709) was diluted with 0.9% saline to a concentration of 1 mg/mL. Normal Goat isotype-matched IgG (R&D, AB-108-C) was used as control. Tie2-Cre;tdTomato mice were fed with high fat diet starting at 8 weeks old and weighed weekly. Aliquots of 50μg of anti-mIL-17B IgG or control IgG were prepared and administered in a blinded fashion every 72 hours by intraperitoneal injection from 14 weeks old and analyzed 48 hours after the last injection at 20 weeks old.

#### Microscopic analysis

To measure microvascular complexity, Tie2-Cre;tdTomato expressing vessels were used for the analyses of vessel volume, vessel length, and junction point. Vessel volume was measured by Imaris software with automated “Add surface” function. Volume of small particle less than 30μm^3^ was subtracted to eliminate the background interference. The masked volume that created by Imaris was identified as vessel volume. Vessel length and junction point were analyzed by AngioTool. The parameters for AngioTool measurement were set as “Diameter 5–40”, “Intensity 40–255” and “Particles less than 10000”.

Analysis of the spatial distribution of stroke-responsive OPCs was performed as follows. The boundary of increased PDGFR-α-+ cells and the loss of GST-π-+ cells was identified in each of three sections per animal (*n* = 3 animals/group). Using Imaris software, the x,y,z position of each PDGFR-α-+ cell relative to the user defined center point (x = 0, y = 0, z = 0) of the elliptical stroke region was determined using the automated “Add Spots” function. Individual cell areas were generated by Imaris with “Add Surface” function. Because the z-axis was limited (10 μm), a two-dimensional grid analysis was performed using a 2D modification of the previously reported 3D spatial density estimator using a smoothing parameter of *k* = 8. The local cell density in each position within the overlaid grid is compared statistically as previously described. Therefore, a *p*-value map is generated for each position in the grid and thresholded (*p*<0.05) to reveal regions with significant density differences. The size of PDGFRα+ OPC was measured individually by Imaris with automated “Add surface” function. Voxel of small particle less than 800 was subtracted to eliminate the background interference. The masked area of PDGFRα+ OPC that created by Imaris was identified as the size of OPC. OPC-vessel distance was measured by Imaris with “Add spot” function. For PDGFRα+ OPC location, nucleus with Dapi staining was used as a reference. The distance of Tie2-Cre;tdTomato vessel to PDGFRα+ OPC was measured with the function of “Spot to Spot closest distance”.

The levels of CXCL5/IL-17Rb in IgG/IL-17B treated mice white matter were measured by Imaris “Coloc” function. The percentages of CXCL5/IL-17Rb that colocalized with Tie2cre;tdTomato positive vessels were measured as voxel areas. For GLUT-1/CXCL5 colocalization measurement, GLUT-1 positive vessels were masked by Imaris with “Add surface” to create new GLUT-1 and CXCL5 channels. The percentages of GLUT-1/CXCL5 colocalization in new channels were measured as voxel areas by Imaris “Coloc” function.

#### Immunohistochemistry for human brain samples

Case selection was made from a subset of 950 UC Davis ADC Neuropathology Core samples based on *a priori* selection criteria: low Braak and Braak scores, at least 80% with some pathologic evidence of cerebrovascular disease sufficient to cause dementia. The most recent cases available were selected based on the selection criteria. Immunohistochemistry was performed using formalin (Medical Chemical Corporation, 575A) fixed paraffin embedded tissue sections cut at 6μm. Sections where placed on positive charged slides (Fisherbrand, 12-550-15) then incubated overnight at 60°C. De-paraffinization was accomplished with three 5min xylene (Fisher Scientific, X3P) washes. The samples were rehydrated with graded concentrations of alcohol (American MasterTech, ALREACS) diluted with deionized water. Endogenous peroxidase was blocked with a 3% solution of hydrogen peroxide (Fisher Scientific, H325–500) 20min incubation. Heat-induced epitope retrieval used a citrate buffer (BioCare Medical, CB910M). The slides incubated in the buffer at 90°C for 45min. Blocking used 2.5% normal horse serum (Vector, S2012) for 60min. Antigen specificity was elucidated by incubating the slides for 90min in CXCL5/6 (1:100, Abcam, ab198505). Primary antibody detection was amplified with a 45min incubation using a secondary antibody (Vector, MP-7401). A 5 second counterstain used hematoxylin (Richard Allan Scientific, 7221). The samples were dehydrated with graded alcohols and three xylene washes before being coverslipped.

### QUANTIFICATION AND STATISTICAL ANALYSIS

The number of animals used in each experiment is listed in the Results section. Vessel densities and oligodendrocyte population cell counts as a fraction of total cells were determined by averaging counts from 5 fields of view (FOV) throughout the corpus callosum across a minimum of three sections 240 μm apart. Per animal averages were generated and significance between groups determined using an unpaired Welch’s t-test (α = 0.05). Measurements of white matter ultrastructural features were determined using 6 FOVs and averaged across animals and compared at the feature level separately using Mann-Whitney U test between groups (α = 0.05). Determination of stroke lesion area was performed by sampling lesion area (*n* = 3–5 40 μm sections) across groups (n = 4/grp) and using the sampled distribution to create bootstrapped area distribution (*n* = 25) representing a full area sampling of the approximate 1 mm lesion created by the stroke model. This area distribution was averaged across animals in each group and compared using a Mann-Whitney U test between groups (α = 0.05). Spatial analysis of stroke-responsive OPCs were determined as above. Cell counts at 28d post-stroke were determined across three sections 240 μm apart with lesion core and edge analyses determined using a two-way ANOVA (α = 0.05) with post-hoc Holm-Sidak test to correct for multiple comparisons. Post-stroke myelination was determined using a Chi-square comparison of distributions. Gene expression differences were determined at the individual gene level using unpaired Welch’s t-test (α = 0.05). CXCL5 values in conditioned media were analyzed using a Krusal-Wallis test with false discovery rate correction. Human serum CXCL5 levels were log_10_ transformed and compared by Mann-Whitney U test. Fazekas scale scores were compared using an ordinal shift Chi-square analysis. Human CXCL5+ vessel segments were compared by two-tailed, one-sample t-test assuming no expression of CXCL5 in non-injured tissue. Unless otherwise stated, all other comparisons were determined using a one-way ANOVA with post-hoc Holm-Sidak test to correct for multiple comparisons. Statistical analysis was performed using GraphPad Prism 7 software. Data are shown as mean ± SEM.

## Supplementary Material

1

2

3

4

## Figures and Tables

**Table 1.** Chemokine-signaling-pathway-related genes differentially expressed in DIO PDGFRα:RiboTAG OPCs

Gene	logFC	Adj. p value	Location in chemokine signaling pathway

ADCY2	0.3783	0.00007	cAMP signaling pathway
ADCY5	0.4331	0.03644	cAMP signaling pathway
PRKACB	0.2698	0.00240	cAMP signaling pathway
CX3CL1	0.6773	0.00000	cytokine-cytokine receptor interaction
GRK3	−0.2817	0.04388	cytokine-cytokine receptor interaction
GRK5	−0.2740	0.01094	cytokine-cytokine receptor interaction
CCL27A	−1.1741	0.00916	cytokine-cytokine receptor interaction
CCR5	1.6514	0.00055	cytokine-cytokine receptor interaction
CCL25	−1.0643	0.04174	cytokine-cytokine receptor interaction
CXCL12	−1.3069	0.02963	cytokine-cytokine receptor interaction
RASGRP2	1.1673	0.01699	diacylglycerol pathway
GNG2	0.3214	0.04134	diacylglycerol pathway
GNB1	−1.4145	0.00731	diacylglycerol pathway
GNB4	−1.9793	0.00019	diacylglycerol pathway
GNB5	−1.3031	0.02824	diacylglycerol pathway
STAT3	−1.7856	0.00147	Jak-STAT signaling pathway
SHC2	−0.3250	0.03276	MAPK signaling pathway
NRAS	−0.4424	0.00585	MAPK signaling pathway
MAP2K1	0.2114	0.01887	MAPK signaling pathway
SOS1	0.5083	0.00089	MAPK signaling pathway
GSK3B	−1.5297	0.00350	PIP-Akt signaling pathway
FOXO3	0.2776	0.01386	PIP-Akt signaling pathway
PIK3CG	1.3907	0.00916	PIP-Akt signaling pathway
PIK3R5	1.3333	0.00095	PIP-Akt signaling pathway
ROCK1	−1.2784	0.03343	regulation of actin cytoskeleton
WASL	−1.8148	0.00012	regulation of actin cytoskeleton
RAC2	1.6040	0.00049	regulation of actin cytoskeleton
PARD3	0.4322	0.03699	regulation of actin cytoskeleton
ELMO1	−1.4219	0.00125	regulation of actin cytoskeleton
PTK2	−0.9568	0.04945	regulation of actin cytoskeleton/diacylglycerol pathway
CRK	0.4503	0.03329	regulation of actin cytoskeleton/diacylglycerol pathway

Differentially expressed genes (FDR < 0.05) from HFD OPCs were filtered for those found in the KEGG Chemokine Signaling Pathway (KEGG Map #04062). Functional enrichment for chemokine signaling was established using a STRING database search (FDR for pathway enrichment = 1.62 × 10^−55^). Gene name, log fold change (logFC), adjusted p value (adj. p value), and location of the gene within the signaling pathway are shown.

**KEY RESOURCES TABLE:** 

REAGENT or RESOURCE	SOURCE	IDENTIFIER

Antibodies		

mouse anti-NF200	Sigma	N4142; RRID:AB_477272
Rabbit anti-GLUT1	Cell Signaling Technologies	73015S
rabbit anti-MBP	Sigma	MAB381
goat anti-PDGFR*α*	Neuromics	RRID:AB_2737233
mouse anti-HA	Biolegend	901501; RRID:AB_2565006
rabbit-Gst-*π*	Millipore	AB8902; RRID:AB_92368
rat anti-IL-17Rb	Santa Cruz Biotech	sc-73969; RRID:AB_2296014
rat anti-CXCL5	R&D Systems, Inc.	MAB433-100; RRID:AB_2086587
Rabbit anti-human CXCL5/6	Abcam	ab198505
Anti-IL-17B Blocking Antibody	R&D Systems, Inc.	AF1709; RRID:AB_354946

Bacterial and virus strains		

Stbl3 E.coli cells	ThermoFisher	C737303
pCDH-FLEX-CXCL5-T2A-GFP	This paper	N/A
pCDH-FLEX-GFP	This paper	N/A

Biological samples		

UCLA ASPIRE Study Biospecimens	Altendahl et al., 2022^42^	N/A
UC Davis ADRC Study Biospecimens	Altendahl et al., 2022^42^	N/A

Chemicals, peptides, and recombinant proteins		

10% kCal from fat mouse chow	Research Diets	D12450J
60% kCal from fat mouse chow	Research Diets	D12492
Tamoxifen	Sigma	85256
L-N^5^-(1-Iminoethyl) ornithine, dihydrochloride	Calbiochem (Sigma)	400600
RNasin	Promega	N2115
Superasin	Thermofisher Scientific	AM2696
NP-40	AG Scientific	N-2366–10X5ML
Diethyl Pyrocarbonate	AG Scientific	D-2569–25ML
Protein G beads	Pierce	88847
IL-17 ligands (A-E)	R&D Systems, Inc.	317-ILB-050 1248-IB-025/CF 1234-IL-025/CF 1504-IL-025/CF 1258-IL-025/CF
Recombinant Mouse CXCL5	R&D Systems, Inc.	433-MC-025/CF
Phallodin-488	Abcam	ab176753

Critical commercial assays		

CXCL5 Quantikine Elisa Kit	R&D Systems, Inc.	MX000
Custom Luminex Assay	R&D Systems, Inc.	N/A
NucleoSpin^®^ miRNA	Machery-Nagel	740971.50
Custom Nanostring RNA hybridization assay	Nanostring	N/A
TruSeq Stranded Total RNA Kit	Illumina	20020596
Ribo-Zero plus rRNA Depletion Kit	Illumina	20037135
HiFi DNA Assembly Kit	New England Biolabs, Inc.	E5520S
Endotoxin-Free PureLink Plasmid Midiprep Kit	ThermoFisher Scientific	K210014

Deposited data		

Endothelial white matter CFD vs. HFD RNA-seq data	This paper	GSE217356
OPC white matter CFD vs. HFD RNA-seq data	This paper	GSE217356
Biomarker data available via OSF link	This paper	https://doi.org/10.17605/OSF.IO/PSQ53

Experimental models: Cell lines		

Primary human brain microvascular endothelial cells	Cell Systems, Inc.	ACBRI 376
Human embryonic kidney 293 cells	ATCC	CRL-11268

Experimental models: Organisms/strains		

C57Bl6/J DIO Strain	Jackson Labs	C57BL/6J DIO Strain #:380050 RRID:IMSR JAX:380050
C57Bl6/J Control Strain	Jackson Labs	C57BL/6J DIO Control Strain #:380056 RRID:IMSR JAX:380056
Tie2-Cre:RiboTAG Strain	Jackson Labs	B6.Cg-Tg(Tek-cre)12Flv/J Strain #:004128 RRID:IMSR JAX:004128 x B6J.129(Cg)-Rpl22tm1.1Psam/SjJ Strain #:029977 RRID:IMSR JAX:029977
PDGFRa-CreERT^2^:RiboTAG Strain	Jackson Labs	B6N.Cg-Tg(Pdgfra-cre/ERT)467Dbe/J Strain #:018280 RRID:IMSR JAX:018280 x B6J.129(Cg)-Rpl22tm1.1Psam/SjJ Strain #:029977 RRID:IMSR JAX:029977
Tie2-Cre:Ai9-tdT Strain	Jackson Labs	B6.Cg-Tg(Tek-cre)12Flv/J Strain #:004128 RRID:IMSR JAX:004128 x B6.Cg-*Gt(ROSA)26Sor*^*tm9<CAG-tdTomato)Hze*^/J Strain #:007909 RRID:IMSR JAX:007909

Oligonucleotides		

Refer to Table S7 for details	Eurofins Genomics	N/A

Recombinant DNA		

murine CXCL5	Origene	#MR200761
pAAV-FLEX-GFP vector	Addgene	#28304
pCDH-EF1-MCS-copGFP	System Biosciences	CD511B-1
pCR-Blunt II TOPO	ThermoFisher Scientific	K280002
pMDLg/pRRE packing vector	Addgene	#12253
pRSV-REV plasmid vector	Addgene	#12251
pMD2.G envelope vector	Addgene	#12259

Software and algorithms		

Imaris	Oxford Instruments	SCR_007370
Angiotool	National Cancer Institute	SCR_016393
Fiji	Open Source	SCR_002285
Custom Matlab Code	This paper	N/A
EdgeR	Open Source	SCR_012802
Gorilla	Ref	N/A
Enrichr	Open Source	SCR_001575
KEGG Pathway Analysis	Open Source	SCR_012773
STRING	Open Source	SCR_005223
3D Spatial Density Estimator	Open Source	SCR_009578

## Highlights

- Diet-induced obesity (DIO) exacerbates the response to subcortical ischemic stroke
- DIO drives abnormal immune signaling in white matter blood vessels in mouse and humans
- IL-17Rb activation drives endothelial CXCL5 expression bringing OPCs to vessels
- Circulating serum levels of IL-17B and CXCL5 are biomarkers for white matter injury
