# Supplementary material for: IL-17/CXCL5 signaling within the oligovascular niche mediates human and mouse white matter injury
Source: Cell Rep. Author manuscript; Available in PMC 2023 Mar 20. (PMC10026849; doi:10.1016/j.celrep.2022.111848)
Supplement: 1 [file NIHMS1860004-supplement-1.pdf]

Cell Reports, Volume *41*

Supplemental information

IL-17/CXCL5 signaling within the oligovascular niche mediates human and mouse white matter injury

Guanxi Xiao, Rosie Kumar, Yutaro Komuro, Jasmine Burguet, Visesha Kakarla, Ida Azizkhanian, Sunil A. Sheth, Christopher K. Williams, Xinhai R. Zhang, Michal Macknicki, Andrew Brumm, Riki Kawaguchi, Phu Mai, Naoki Kaneko, Harry V. Vinters, S. Thomas Carmichael, Leif A. Havton, Charles DeCarli, and Jason D. Hinman

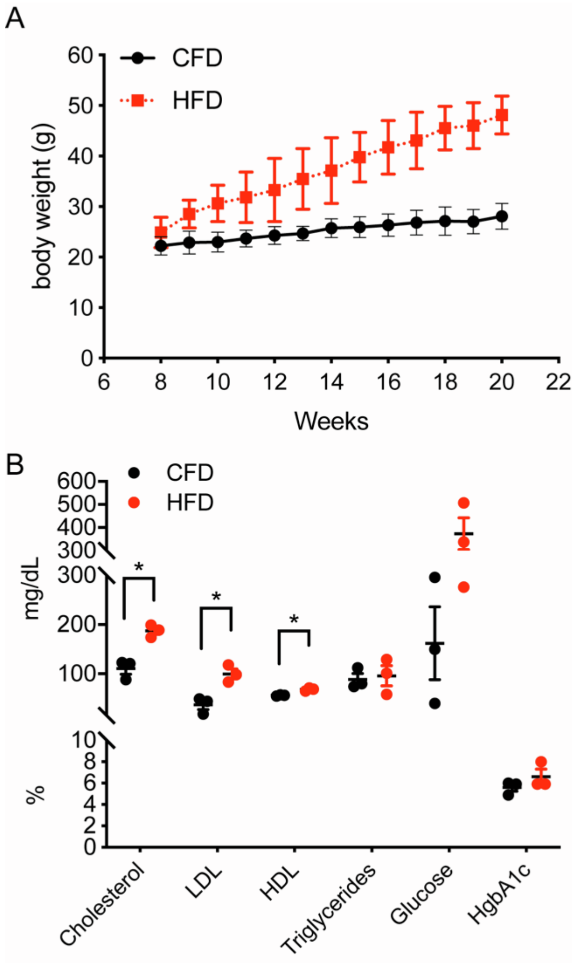

Figure S1. High fat diet induces obesity and metabolic syndrome in mice, Related to Figure 1. Week by week body weight measurements of C57BL/6Jfed ad lib with CFD (black) or HFD (red) with final average 84.0% weight gain compared to 21.7% in CFD mice (**p=0.0005; n=5/grp) (A). Serologic testing of C57BL/6Jmice on CFD or HFD (n=3) for total cholesterol (110.7±2.6vs. 187.3±2.5; *p=0.023), LDL(37.0 ±2.4 vs. 99.7±2.0; *p=0.043), glucose (162.0±6.5vs. 373.3±4.2) and HgbA1c (5.6 ±0.5vs. 6.6±0.2)(B). Data are mean ± SEM.

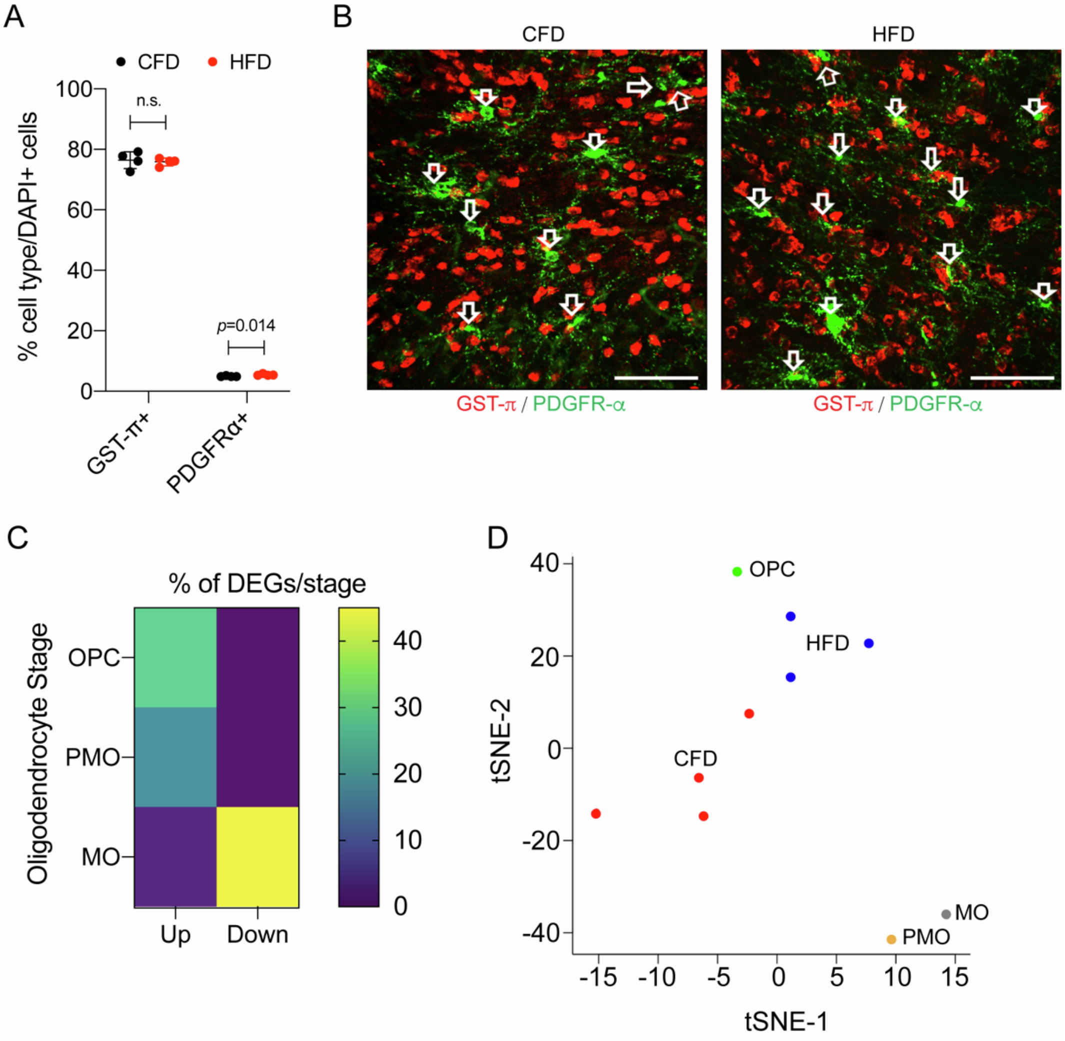

Figure S2. DIO causes white matter disruption and favors immature oligodendrocytes, Related to Figure 1. Oligodendrocyte cell type counts within subcortical white matter in animals on CFD vs. HFD (A). Representative microscopy images of subcortical white matter from animals on CFD (left) vs. HFD (right) immunostained for the mature oligodendrocyte marker GST-π (red) and oligodendrocyte progenitor cell marker PDGFR-α (green). Heatmap of the percentage of differentially expressed genes ($p<0.05$) per oligodendrocyte lineage stage (OPC = oligodendrocyte progenitor cell; PMO = premyelinating oligodendrocyte; MO = myelinating oligodendrocyte) generated by Nanostring Oligodendrocyte Staging Assay (total 120 genes/40 per stage selected from Zhang et al.) for animals on CFD vs. HFD (n=4/grp) showing only OPC and PMO genes were up-regulated by DIO while only MO genes were down-regulated (C). tSNE (t-distributed Stochastic Neighbor Embedding) of Nanostring gene expression for CFD (red) and HFD (blue) animals using reference profiles of OPCs, pre-myelinating oligodendrocytes (PMO), and myelinating oligodendrocytes (MO) generated from reference marker gene expression profiles (1) shows that gene expression profiles of animals on HFD cluster more closely with OPCs while those from animals on CFD have equal representation of oligodendrocyte subtypes and thus cluster independently (D). Scale bar = 50 μm (B).

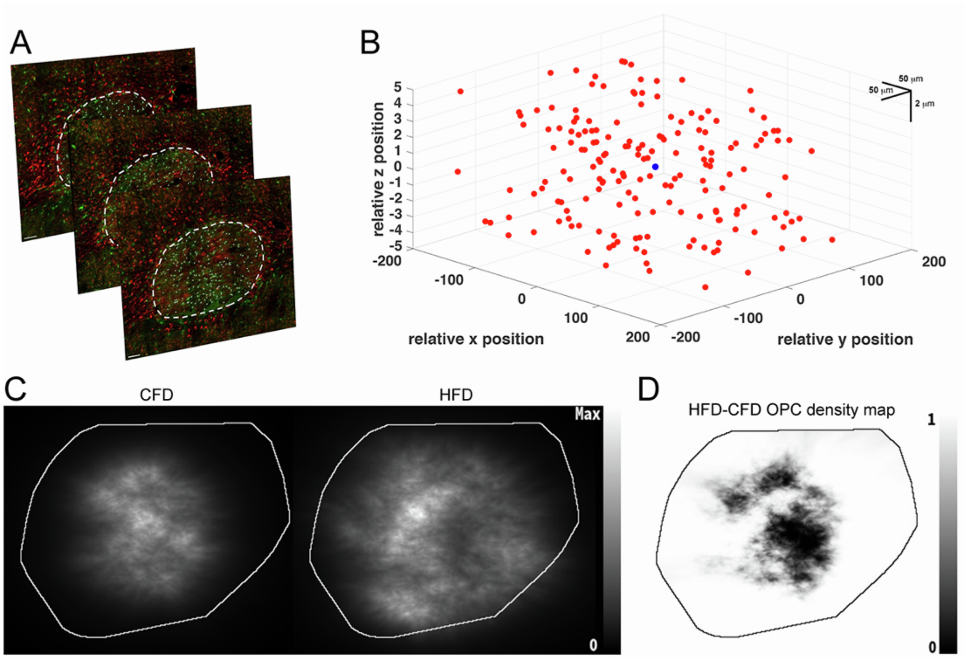

Figure S3. Quantitative spatial mapping of OPCs in white matter stroke lesions, Related to Figure 2. Confocal z-stacks through the white matter stroke lesion were used to identify stroke-responsive PDGFRα+ OPCs (green) within the lesion core where GST-π+ mature oligodendrocytes (red) were lost due to focal ischemia (11 steps of Z-stacks per image) (A). Spatial coordinates of each cell (x,y,z in µm) relative to the center point of the elliptical stroke lesion (blue dot) were used to generate spatial maps of stroke-responsive OPCs (red) within each lesion section (9 sections/group; 3028 cells spatially measured). Representative coordinate map of stroke-responsive OPC localization from one lesion (B). Average OPC spatial density maps were generated in 2D for animals on CFD (left, n=3) and those on HFD (right, n=3) as described (J. Burguet et al Pattern Recogn Lett, 2011). Vertical gray bar represents cellular density with max density value=3.97×10^{-3}cells/µm^2. Curve in white: ROI contour (C). Map of p-values for differences in stroke-responsive OPC densities between animals on CFD and HFD. Vertical gray bar represents p-value. Low p-values (<0.05; black) correspond to regions with significantly more cells in the control group, and high p-values (>0.95; white) to regions with significantly more cells in the HFD group (D).

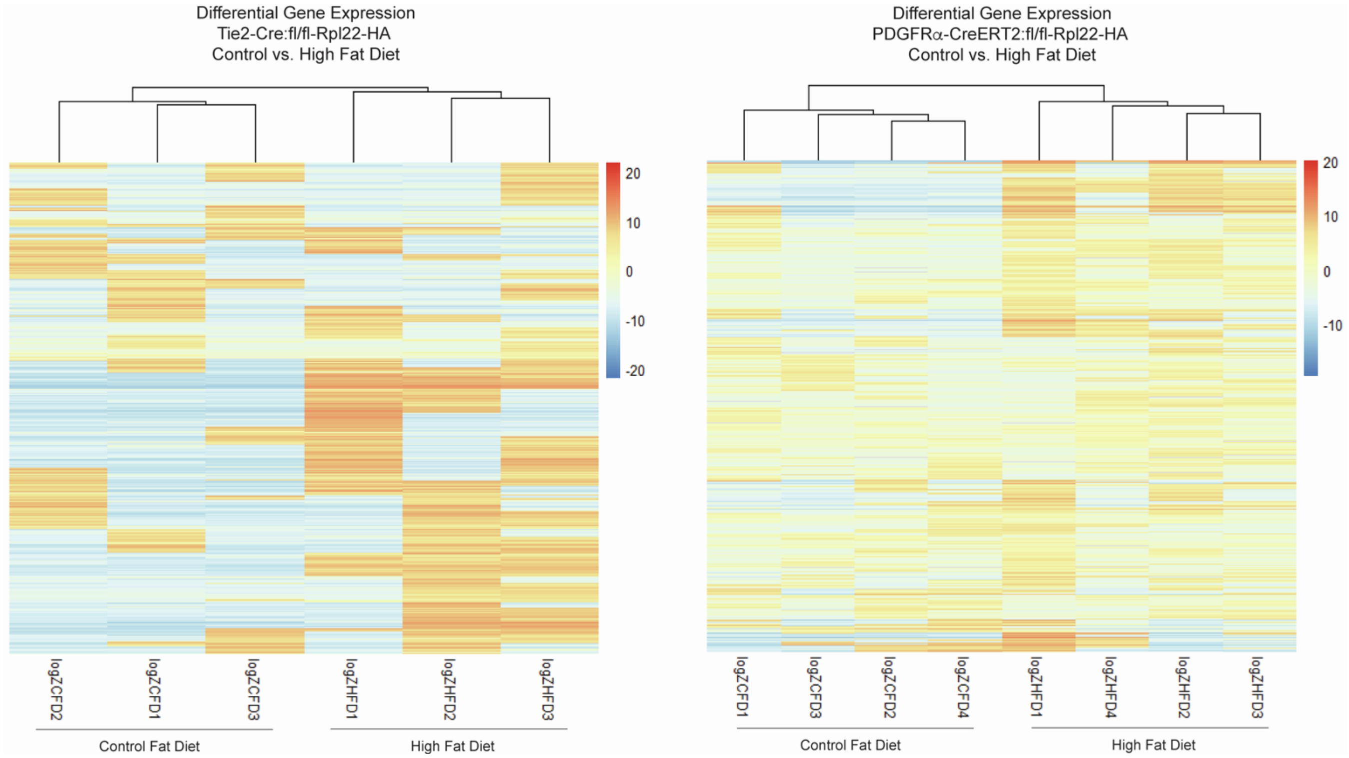

Figure S4 – Endothelial and oligodendrocyte progenitor cell differentially expressed genes in DIO, Related to Figure 3. Heatmaps of RNA-Seq expression coefficients computed for expressed genes between control diet (CFD) and high-fat diet (HFD) in Tie2-Cre:fl/fl-Rpl22-HA (endothelial) and PDGFRα-CreERT^{2}:fl/fl-Rpl22-HA (oligodendrocyte progenitor cell) mice. Columns represent individual biologic replicates.

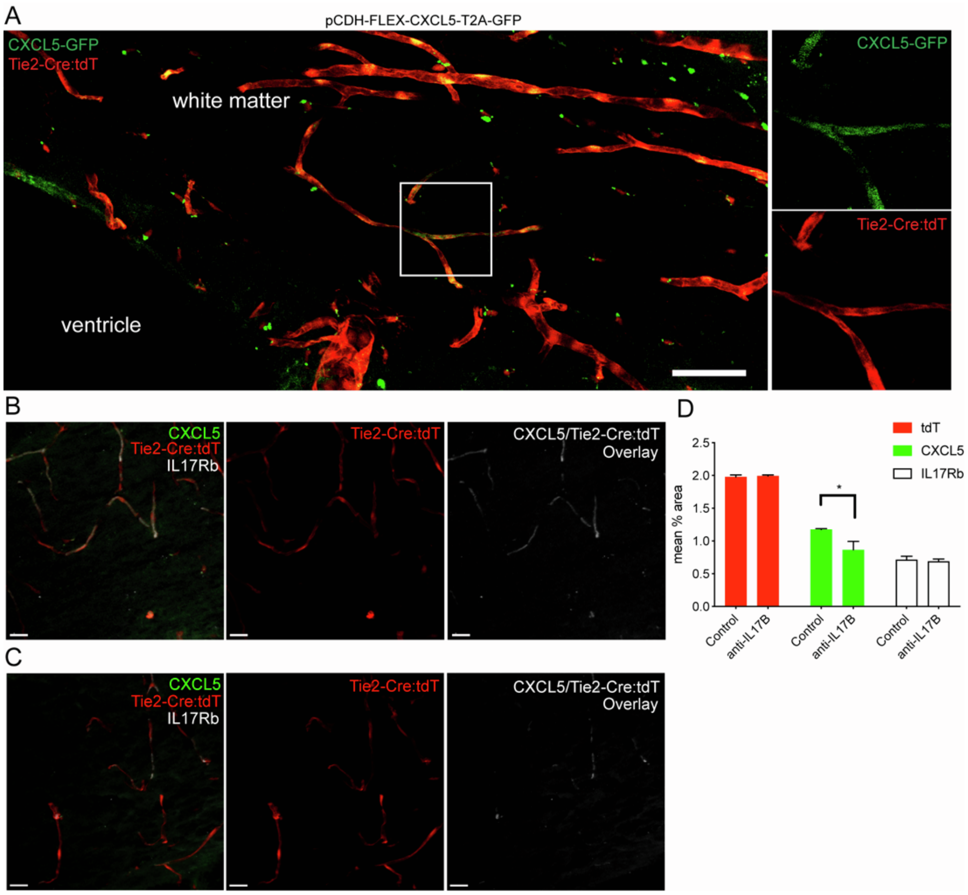

Figure S5. Modulation of CXCL5 expression in white matter vasculature using combined transgenic and viral transduction or anti-IL-17B IgG, Related to Figure 5. Animals on control fat diet underwent viral transduction into the white matter with pCDH-FLEX-CXCL5-T2A-GFP lentivirus (shown) or control pCDH-FLEX-GFP lentivirus. Viral particles at a titer of 10^8 were injected to white matter in Tie2-Cre;tdTomato mice at 14 weeks old and analyzed at 20 weeks of age. After 6 weeks of viral transduction, CXCL5-T2A-GFP (green)was highly colocalized with Tie2-Cre;tdTomato+vessels (red) within the region of viral targeting in white matter (insets, A). To reduce DIO-induced endothelial expression of CXCL5, function blocking anti-IL-17B IgG or control isotype-matched IgG (50 µg) was administered q3d for 6 weeks in Tie2-Cre;tdTomato animals on HFD beginning at 14 months of age. At 20 weeks of age, immunolabeling for CXCL5 (green) and IL17Rb (white) was performed in animals receiving control IgG (B) or anti-IL-17B IgG (C). In white matter tdT+ vasculature, both CXCL5 and IL17Rb were colocalized with Tie2-Cre;tdTomato+ vessels. Compared to animals administered control IgG, mean % area occupied by immunolabeling of CXCL5 was significantly reduced in animals administered anti-IL-17B IgG (1.17 vs. 0.86; *p=0.0067; n=4) (D). Endothelial expression of IL17Rb (mean % area) was not significantly different between control IgG and anti-IL-17B IgG indicating that receptor expression was not altered by anti-IL17B IgG administration. Scale bars = 50 µm (A); 20 µm (B-C). Data are mean ± SEM.

Supplemental Table 4. Expression of IL-17 family member genes in DIO white matter endothelia, Related to Figure 3.

IL-17 family member	Log-fold change (HFD vs. CFD)	*p*-value	False discovery rate
Il17Ra	0.999	0.470	0.820
**Il17Rb**	**8.835**	**0.001**	**0.090**
Il17Rc	-2.695	0.303	0.695
Il17Rd	-4.038	0.043	0.397
Il17Re	2.918	0.322	0.713
Il17a	not detected	not detected	not detected
Il17b	0.000	1.000	1.000
Il17c	not detected	not detected	not detected
Il17d	-0.318	0.805	1.000
Il17f	not detected	not detected	not detected

Supplemental Table 5. Descriptive characteristics of post-mortem cohort, Related to Figure 6.

Age	Sex	Ethnicity	Clinical Syndrome	Pathological Diagnosis	BRAAK	PMI	Any positive vessel	% positive vessel segments (# positive)
75	M	Hispanic	No cognitive impairment	Normal Brain	2	49.5	Yes	35.3% (6)
83	M	White	No cognitive impairment	Possible AD	2	30	Yes	60.0% (9)
96	F	White	No cognitive impairment	Normal Brain	2	6.9	Yes	94.4% (17)
86	F	White	Questionable cognitive impairment	Vascular disease, infarcts only	1	-	No	0% (0)
84	F	White	No cognitive impairment	Normal Brain	1	10.9	Yes	100% (24)
72	M	White	No cognitive impairment	Vascular disease, combined lacunar and large infarctions, cerebrovascular disease	1	4	Yes	50.0% (9)
89	M	Hispanic	Dementia	Vascular disease, multiple lacunes	2	69	Yes	71.4% (15)
92	F	White	Dementia	Vascular disease, multiple lacunes	3	7	Yes	50.0% (7)
90	F	Asian	Dementia	Vascular disease, Binswangers disease. Pathological findings of white matter vasculopathy	1	40	No	0% (0)
92	F	Hispanic	Dementia	Limbic sclerosis, entorhinal neocortex	3	31	Yes	84.6% (11)

Supplemental Table 6. Mixed effects models of CXCL5 as a prognostic biomarker for VCID, Related to Figure 6.

Mean Longitudinal Executive Function Performance			
Predictor	Estimate	*CI*	*p-value*
Intercept	-0.0163	-0.9189 to 0.8862	0.97
Age	-0.0171	-0.0282 to -0.0060	0.0028
Education	0.0661	0.0450 to 0.08720	<0.0001
Sex	-0.0851	-0.1661 to -0.0041	0.04
Dementia at baseline	-0.5117	-0.8964 to -0.1270	0.01
MCI at baseline	-0.0986	-0.3284 to 0.1313	0.40
Baseline CXCL5	$4.61x10^{-5}$	$8.66x10^{-5}$ to $-5.68x10^{-5}$	0.026

Supplemental Table 7. Oligonucleotides used in the study.

qPCR Primers		
Gene Name	Forward primer (5’>3’)	Reverse Primer (5’>3’)
IL17Rb	GGACAGGTTGTGCTTTGCTCA	GAAAGGACGTCTTCGTGCTC
Cxcl5	GAAAGCTAAGCGGAATGCAC	GGGACAATGGTTTCCCTTTT
Tnfrsf10b	GTCAGAAGGGAACTGCAAGC	GCATCGACACACCGTATTTG
Itgb3	TGACATCGAGCAGGTGAAAG	GAGTAGCAAGGCCAATGAGC
RN18s	GTAACCCGTTGAACCCCATT	CCATCCAATCGGTAGTAGCG
Cloning Primers		
T2A-copGFP	GTTATACTAGGGATCCCCCCCGCGAGATCCGGTGGAGCCG	CAAGGAAAACGCGGCCGCTGAGGGCAGA
mouse CXCL5	GTTATACTAGGGATCCCCCCCCTGCAGATGCATTTATTGAACACTGGCCGTTCTTTC	CCTATACGAAGTTATACTAGATGAGCCTCCAGCTCCGC
